# The highly dynamic satellitomes of cultivated wheat species

**DOI:** 10.1093/aob/mcae132

**Published:** 2024-08-30

**Authors:** Ana Gálvez-Galván, Manuel A Garrido-Ramos, Pilar Prieto

**Affiliations:** Plant Breeding Department, Institute for Sustainable Agriculture, Agencia Estatal Consejo Superior de Investigaciones Científicas (CSIC), Avda. Menéndez Pidal, Campus Alameda del Obispo s/n, 14004 Córdoba, Spain; Departamento de Genética, Facultad de Ciencias, Universidad de Granada, Avda. Fuentenueva s/n, 18071 Granada, Spain; Plant Breeding Department, Institute for Sustainable Agriculture, Agencia Estatal Consejo Superior de Investigaciones Científicas (CSIC), Avda. Menéndez Pidal, Campus Alameda del Obispo s/n, 14004 Córdoba, Spain

**Keywords:** Durum wheat, satellite DNA, satellitome, TEs, centromeres, subtelomeres, chromosome recognition, homologous pairing, genome evolution, cereal evolution, *Triticum* evolution

## Abstract

**Background and Aims:**

Durum wheat, *Triticum turgidum*, and bread wheat, *Triticum aestivum*, are two allopolyploid species of very recent origin that have been subjected to intense selection programmes during the thousands of years they have been cultivated. In this paper, we study the durum wheat satellitome and establish a comparative analysis with the previously published bread wheat satellitome.

**Methods:**

We revealed the durum wheat satellitome using the satMiner protocol, which is based on consecutive rounds of clustering of Illumina reads by RepeatExplorer2, and we estimated abundance and variation for each identified satellite DNA (satDNA) with RepeatMasker v4.0.5. We also performed a deep satDNA family characterization including chromosomal location by fluorescence *in situ* hybridization (FISH) in durum wheat and its comparison with FISH patterns in bread wheat. BLAST was used for trailing each satDNA in the assembly of durum wheat genome through NCBI’s Genome Data Viewer and the genome assemblies of both species were compared. Sequence divergence and consensus turnover rate between homologous satDNA families of durum and bread wheat were estimated using MEGA11.

**Key Results:**

This study reveals that in an exceedingly short period, significant qualitative and quantitative changes have occurred in the set of satDNAs of both species, with expansions/contractions of the number of repeats and the loci per satellite, different in each species, and a high rate of sequence change for most of these satellites, in addition to the emergence/loss of satDNAs not shared between the two species analysed. These evolutionary changes in satDNA are common between species but what is truly remarkable and novel is that these processes have taken place in less than the last ~8000 years separating the two species, indicating an accelerated evolution of their satDNAs.

**Conclusions:**

These results, together with the relationship of many of these satellites with transposable elements and the polymorphisms they generate at the level of centromeres and subtelomeric regions of their chromosomes, are analysed and discussed in the context of the evolutionary origin of these species and the selection pressure exerted by humans throughout the history of their cultivation.

## INTRODUCTION

Wheat is the most cultivated cereal and has been linked to the economic and cultural history of humans. Specifically, durum wheat [*Triticum turgidum* L. subsp. *durum* (Desf.) Husn.] is the tenth most important crop worldwide and ~50 % of the world’s production of this species is in the Mediterranean area (Martínez-Moreno *et al.*, 2022). Durum wheat is renowned for its high protein content and gluten strength, which make it ideal for pasta production. Semolina is a key ingredient in making pasta, giving its characteristic texture and flavour. The Mediterranean diet, which emphasizes whole grains, vegetables, fruits and olive oil, often incorporates pasta made from durum wheat semolina as a staple food. Human progress and the development of wheat agriculture have gone hand in hand.

Among crop species, wheat was one of the earliest to be domesticated in the Fertile Crescent. Approximately 800 000 years ago, the first hybridization event took place between two diploid species, the male donor of the A subgenome, possibly a species related to *Triticum urartu* (2*n* = 2*x* = 14; genome AA) (Dvořák *et al.*, 1993; Baum and Grant Bailey, 2004), and the female donor of the B subgenome, a species closely related to *Aegilops speltoides* (2*n* = 2*x* = 14; genome BB) (Li *et al.*, 2022), leading to the tetraploid wild emmer wheat, *Triticum turgidum* subsp. *dicoccoides* (Körn. ex Asch. & Graebn.) Thell. (2*n* = 4*x* = 28; genome BBAA). The domestication of this species gave rise to domesticated emmer, *T. turgidum* L. subsp. *dicoccon* (Schrank) Thell. Schübl (2*n* = 4*x* = 28; genome BBAA) (Levy and Feldman, 2022; Li *et al.*, 2022). Durum wheat seems to have originated from a selection of hulled tetraploid wheat genotypes (like cultivated emmer). There are two possible early free-threshing wheat species, namely the extinct tetraploid *T. turgidum* subsp*. parvicoccum* (2*n* = 4*x* = 28; genome BBAA) (Feldman *et al.*, 2001) and a more recent tetraploid wheat known as rivet wheat [*Triticum turgidum* L. subsp. *durum* (Desf.) Husn.] (2*n* = 4*x* = 28; genome BBAA). Rivet wheat is similar to durum wheat but has softer grains and is more tolerant to cold (Martínez-Moreno *et al.*, 2022). Domesticated emmer wheat is the ancestor of the contemporary cultivated durum wheat, *Triticum turgidum* subsp. *durum* (2*n* = 4*x* = 28; genome BBAA) (Feldman *et al.*, 2001). Bread wheat (*Triticum aestivum* L. subsp. *aestivum*) (2*n* = 6*x* = 42; genome BBAADD) originated from spontaneous hybridization processes between tetraploid wheat species that were cultivated by human communities in the past with the coetaneous wild species *Aegilops tauschii* (2*n* = 2*x* = 14; genome DD). This hybridization process took place over thousands of years and resulted in a polyploidization process, which has been reviewed and discussed on several occasions (McFadden and Sears, 1946; Dvorak *et al.*, 1998; Matsuoka, 2011; Brenchley *et al.*, 2012; Deng *et al.*, 2013; Wang *et al.*, 2013). Human genetic selection over generations has resulted in modern cultivars of durum and bread wheat. Each species has distinct characteristics associated with their agronomic traits, grain quality and environmental adaptation. Bread wheat is widely distributed worldwide and adaptable to different environments. The D subgenome contributes to its adaptability (Mastrangelo and Cattivelli, 2021).

We are studying the genomes of two wheat species in the context of breeding to analyse their genome evolution and identify DNA sequences that could be useful markers of reproductive biological processes such as meiosis, which generates the gametes in organisms with sexual reproduction. Both bread and durum wheat lines used in this work have been widely used for decades in a breeding context. For example, several alien genetic introgressions (disomic addition and substitution lines of rye, wild and cultivated barley species) have been developed in *T. aestivum* and *T. turgidum* (Islam *et al.*, 1978, 1981; Miller *et al.*, 1982; Calderón *et al.*, 2012), development of new species (*Tritordeum*, an amphyploid between the wild barley *Hordeum chilense* Roem. et Schult. and *T. aestivum* or *T. turgidum*) (Martín and Chapman, 1977; Martin *et al.*, 1995), the development of the first bread wheat whole genome sequencing analysis (https://www.ncbi.nlm.nih.gov/datasets/genome/GCF_018294505.1/) and numerous meiosis studies (Riley and Chapman, 1958; Calderón *et al.*, 2014; Aguilar and Prieto, 2020), among many others. In this context, we have previously characterized and analysed the bread wheat satellitome [i.e. the set of all satellite DNAs (satDNAs) of its genome] (Gálvez-Galván *et al.*, 2024). We have now analysed the durum wheat genome and we include here a comparative analysis of both bread and durum wheat satellitomes. This analysis demonstrates a high evolutionary dynamism of wheat satDNAs in an extremely short period in which it has probably played an important role in the evolutionary processes that led to the origin of these species (hybridization and polyploidization) as well as their domestication and extensive artificial selection, to which should be added the role of mobile elements, some of which would have given the rise in the past to the satDNAs analysed here.

## MATERIALS AND METHODS

### Plant material and growing conditions

Tetraploid (durum) wheat *Triticum turgidum* L., cv. Capelli (2*n* = 4*x* = 28) was used in this work to perform genomic and cytogenetic analyses. Seeds were incubated for germination on wet filter paper in Petri dishes in the dark at 25 °C for 2 d. Then, seeds were moved to pots and full-grown at 24 ± 2 °C in the glasshouse under a long-day photoperiod.

### Satellitome analysis

Genomic DNA (gDNA) was isolated from durum wheat leaves using the CTAB procedure (Murray and Thompson, 1980) with some modifications (Hernández *et al.*, 2001). DNA concentration and quality were measured with a NanoDrop1000 spectrophotometer (NanoDrop Technologies, USA).

Next generation sequencing was carried out at Macrogen Inc. (MacrogenInc., Seoul, Korea) based on Illumina NovaSeq 6000 150PE (2 × 151 bp), yielding about 20 Gb (~1.7× coverage) data. Raw sequence data were deposited in the SRA-GenBank database in the BioProject PRJNA1036624.

We searched for satDNA sequences using the satMiner protocol (Ruiz-Ruano *et al.*, 2016), which is based on consecutive rounds of clustering of Illumina reads by RepeatExplorer 2 (RE2) (Novák *et al.*, 2013, 2020), using a subset of reads (2 000 000 per library), and subsequent filtering of the already assembled reads using DeconSeq (Schmieder and Edwards, 2011). RE2 executes an integrated version of the TAREAN tool (Novák *et al.*, 2017), which performs automated identification of satDNA repeats based on the topology of their cluster graphs. We first performed a quality trimming with Trimmomatic (Bolger *et al.*, 2014) and randomly selected 2 × 2000 000 Illumina reads with SeqTK (https://github.com/lh3/seqtk), to run RE2 with default options. Cluster graphs with circular shapes were selected with TAREAN, generating a consensus monomer sequence for each satDNA cluster. We filtered out those reads showing homology with the already clustered contigs and the already identified satDNA using DeconSeq. We then selected a new set of 2 × 2000 000 reads from the filtered libraries that were clustered with RE2 in a second round. This allows the detection of satDNAs poorly represented in the raw reads. We repeated the filtering using the clusters in the second round and selected 2 × 2000 000 reads for three additional rounds. Additional rounds of clustering and filtering have been shown to be highly successful as this allows the detection of satDNAs which, due to their low abundance, had gone unnoticed because their signals were masked by those of highly abundant elements (Ruiz-Ruano *et al.*, 2016). After multiple iterations, we performed a similarity search among the sequences with RepeatMasker (Smit *et al.*, 2015) using a custom python script (https://github.com/fjruizruano/ngs-protocols/blob/master/rm_homology.py).

To estimate abundance and divergence for each identified satDNA, we aligned 2 × 10 million randomly selected read pairs to the consensus sequences in the resulting satDNA database, using RepeatMasker v4.0.5 (Smit *et al.*, 2015) with a publicly available script (https://github.com/fjruizruano/satminer/blob/master/repeat_masker_run_big.py). We used the calcDivergenceFromAlign.pl built-in tool of RepeatMasker to obtain a histogram of the Kimura two-parameter (K2P) divergence for each element. Next, we transformed the abundance values to express them as genome proportions by dividing the number of aligned nucleotides by the total number of nucleotides in the selection of 20 million reads.

The EMBOSS suite of bioinformatics tools (Rice *et al.*, 2000) was used for basic analyses of every satDNA family (Gálvez-Galván *et al.*, 2024). A fine search of these satellite sequences in the durum wheat genome was carried out using the Basic Local Alignment Search Tool (BLAST) trailing the genome assembly Svevo.v1 assembly of the *T. turgidum* subsp. *durum* genome through NCBI’s Genome Data Viewer (GDW) to identify the locations of each satDNA family within the durum wheat genome (https://www.ncbi.nlm.nih.gov/genome/gdv?org=triticum-turgidum&group=bop-clade). Similarly, we searched some satDNAs in the genome assemblies of *T. urartu*, *T. monococcum*, *A. speltoides* and *A. tauschii* and different accessions of *T. aestivum*. In addition, we searched for homologies with transposable elements with RepeatMasker (Smit *et al.*, 2015) with ‘no_low’ and ‘no_is’ options.

Finally, we searched for homology between *T. turgidum* and *T. aestivum* satellitomes (Gálvez-Galván *et al.*, 2024) with the rm_homology script (Ruiz-Ruano *et al.*, 2016) that makes all-to-all alignments with RepeatMasker v4.0.5 (Smit *et al.*, 2015). For homologous satellites between the two species, we performed pairwise alignments using ClustalX (Thompson *et al.*, 1997) which were manually reviewed. Then, sequence divergence between satDNA families of *T. turgidum* and *T. aestivum* was calculated following the K2P method (Kimura, 1980), using MEGA11 (Tamura *et al.*, 2013). A consensus turnover rate (CTR) was calculated using the equation CTR = *K*/2*T*, where *T* = divergence time between species and *K* = K2P distance (Camacho *et al.*, 2022). For divergence time, we considered that the origin of *T. aestivum* from *T. turgidum* × *A. tauschii* was ~8000 years ago (McFadden and Sears, 1946; Dvorak *et al.*, 1998; Wang *et al.*, 2013).

### Cytogenetic validation of satDNAs

To obtain DNA sequences for cytogenetic validation of satDNA families, PCR was used to amplify the different satDNAs. We designed the primers using the Primer-BLAST software available at NCBI (https://www.ncbi.nlm.nih.gov/tools/primer-blast/index.cgi?LINK_LOC=BlastHome) (Supplementary Data Table S1), except for the primers for satellites shorter than 80 bp, which were designed by hand. The absence of secondary structures in the sequences of the primers was tested using OligoAnalyzer (https://eu.idtdna.com/calc/analyzer). PCR conditions were different depending on the length of the monomers (longer or shorter than 80 bp). That is, for longer than 80 bp: 94 °C for 5 min, 35 cycles at 94 °C for 30 s, an annealing step of 42–60 °C (primer-dependent, see Additional file 1) for 30 s and an extension at 72 °C for 1 min plus an extension step at 72 °C for 6 min. We reduced the time of annealing to 10 s for those shorter than 80 bp (Ruiz-Ruano *et al.*, 2016). Finally, satDNAs were labelled as described previously (Gálvez-Galván *et al.*, 2024).

Chromosome spreads and treatments were described in Prieto *et al.* (2001, 2004). Seed roots 1–2 cm long were cut and treated in a colchicine solution (0.05 %, w/v) for 4 h at 25 °C. Roots were fixed in 100 % ethanol/acetic acid, 3:1 (v/v) and stored at 4 °C for *in situ* hybridization experiments. Plants were then grown in a glasshouse under semi-controlled conditions of temperature (25 °C day/15 °C night) and relative humidity (40 %).

Preparation of chromosome spreads was done as previously described (Prieto *et al.*, 2004). We hybridized the same sample with two different satDNAs simultaneously, each one in a different colour as obtaining chromosome spreads is the most challenging step of the whole procedure.

*In situ* hybridization and post-hybridization washes were performed as described previously (Cabrera *et al.*, 2002). We identified the different wheat chromosomes using the repeat sequences pAs1 (Rayburn and Gill, 1986; Cabrera *et al.*, 1995) and GAA (Pedersen *et al.*, 1996; Pedersen and Langridge, 1997). Samples were visualized using a Nikon Eclipse 80i epifluorescence microscope coupled with a CCD camera (Nikon Instruments Europe BV, Amstelveen, The Netherlands). In those cases where brightness and contrast needed to be adjusted, Photoshop 11.0.2 software (Adobe Systems Inc., San Jose, CA, USA) was used.

## RESULTS

### The durum wheat satellitome in the context of bread wheat satellitome

**Fig. 1.**
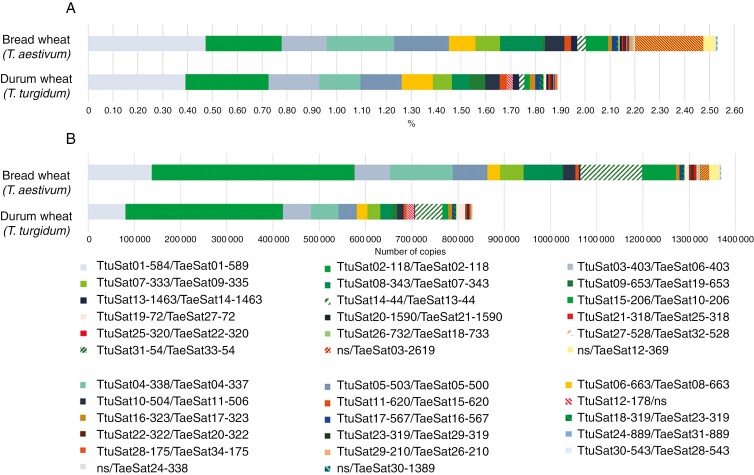
Summary of the composition of the repetitive fraction (satellitome) originating from individual clustering analysis in durum and bread wheat. (a) Percentage of total satellitome according to the genome size; (b) number of copies.

We have identified 31 satDNAs on the cultivated durum wheat library (Table 1). Supplementary Data Fig. S1 shows the consensus sequences for each satDNA family. All but one of these satDNA families were identified previously in the genome of the bread wheat *T. aestivum* cv. Chinese Spring (CS) and they share features such as repeat length, AT content, sequence organization, intraspecific variation and the grouping in superfamilies (satDNAs families that probably derived from a common ancestor satDNA) (Tables 1 and 2; Fig. 1) (Gálvez-Galván *et al.*, 2024). We also found that several of these satDNAs showed homology to other satDNAs previously described both in wheat lines and in other Poaceae (Table S2) as well as to transposable elements (TEs) (Table S3). However, the relative abundance of each satDNA family differs between the two species. Collectively, all 31 satDNAs represent ~1.89 % of the genome of *T. turgidum* (Table 1). That is, assuming a genome size of this species of about 12 054 Mbp (Bennett and Smith, 1976), this percentage represented about 228 Mbp and a total of ~829 604 repeat copies (Table 2). Contrasting these values, 2.53 % of the bread wheat genome is composed of satDNA and, having a bigger genome, this percentage represents about 429 Mbp and 1 368 939 satDNA repeats (Table 2). That is, there has been an increase in the amount of satDNA in the CS bread wheat genome, due to an increase in the copy number of most of the shared satDNAs (21 specifically, and these are among the most abundant in each genome), and the existence of four additional satDNA families undetected in the durum wheat genome (Tables 1 and 2; Fig. 1) (Gálvez-Galván *et al.*, 2024). However, it is relevant that there are four satellites (TtuSat09-653, TtuSat18-319, TtuSat19-72 and TtuSat28-175) that are more abundant in the durum than in the bread wheat genome and, furthermore, there is a satDNA family, TtuSat12-178, that forms conspicuous fluorescence *in situ* hybridization (FISH) loci terminally in chromosomes 1B and 6B (see below) in *T. turgidum*, but was not found by our satMiner search in *T. aestivum*. Furthermore, PCR assays failed to amplify this satDNA from genomic DNA of wheat bread (not shown).

**Table 1.** Comparison of metrics of different parameters of satDNAs identified in durum wheat and bread wheat.

Satellite name	Length	Abundance (%)	Variation	AT content (%)	SF	FISH	Tae homologous	Abundance (%)	Variation	FISH	Divergence	CTR (×10^−6^)
TtuSat01-589	584	0.3919	0.10	38.0		Dispersed	TaeSat01-584	0.473	0.10	Dispersed	0.02	1.44
TtuSat02-118	118	0.3329	0.12	49.2		Multiple locations	TaeSat02-118	0.305	0.13	Multiple locations	0.03	1.63
TtuSat03-403	403	0.2037	0.21	59.8		Dispersed	TaeSat06-403	0.182	0.22	Dispersed	0.16	9.88
TtuSat04-338	338	0.1670	0.12	63.9	SF-1	Multiple locations	TaeSat04-337	0.271	0.08	Multiple locations	0.08	4.69
TtuSat05-503	503	0.1651	0.24	57.9	SF-2	Dispersed	TaeSat05-500	0.220	0.24	Dispersed	0.02	1.38
TtuSat06-663	663	0.1263	0.07	65.0	SF-3	Subtelomeric	TaeSat08-663	0.107	0.07	Subtelomeric	0.02	1.13
TtuSat07-333	333	0.0764	0.09	33.6	SF-4	Multiple locations	TaeSat09-335	0.100	0.10	Multiple locations	0.08	4.94
TtuSat08-343	343	0.0716	0.09	59.6	SF-1	Multiple locations	TaeSat07-343	0.169	0.07	Multiple locations	0.07	4.22
TtuSat09-653	653	0.0633	0.27	54.2	SF-3	Multiple locations	TaeSat19-653	0.011	0.18	Multiple locations	0.01	0.68
TtuSat10-504	504	0.0583	0.10	61.9	SF-2	Dispersed	TaeSat11-506	0.078	0.12	Dispersed	0.02	1.39
TtuSat11-620	620	0.0273	0.23	61.9		Subtelomeric	TaeSat15-620	0.026	0.25	Multiple locations	0.02	1.33
TtuSat12-178	178	0.0255	0.03	55.6		Subtelomeric	n.s.	n.s.	n.s.	n.s.	n.s.	n.s.
TtuSat13-1463	1463	0.0240	0.19	59.1	SF-6	Centromeric	TaeSat14-1463	0.026	0.18	Centromeric	0.00	0.00
TtuSat14-44	44	0.0217	0.08	70.5		Multiple locations	TaeSat13-44	0.035	0.08	Multiple locations	0.02	1.44
TtuSat15-206	206	0.0215	0.09	61.2	SF-1	Multiple locations	TaeSat10-206	0.089	0.06	Multiple locations	0.05	3.16
TtuSat16-323	323	0.0213	0.11	63.0		Subtelomeric	TaeSat17-323	0.014	0.09	Subtelomeric	0.04	2.59
TtuSat17-567	567	0.0196	0.09	53.6	SF-5	Centromeric	TaeSat16-567	0.020	0.12	Centromeric	0.00	0.22
TtuSat18-319	319	0.0138	0.08	41.7	SF-6	Multiple locations	TaeSat23-319	0.007	0.11	Subtelomeric	0.02	0.99
TtuSat19-72	72	0.0116	0.05	48.6		Subtelomeric	TaeSat27-72	0.004	0.08	Subtelomeric	0.01	0.88
TtuSat20-1590	1590	0.0093	0.06	35.6		Dispersed	TaeSat21-1590	0.009	0.06	Dispersed	0.00	0.00
TtuSat21-318	318	0.0070	0.14	47.2	SF-6	Subtelomeric	TaeSat25-318	0.005	0.16	Multiple locations	0.06	3.68
TtuSat22-322	322	0.0070	0.10	39.8	SF-4	Subtelomeric	TaeSat20-322	0.010	0.16	Subtelomeric	0.02	1.18
TtuSat23-319	319	0.0051	0.11	48.3	SF-6	Multiple locations	TaeSat29-319	0.003	0.10	Multiple locations	0.04	2.62
TtuSat24-889	889	0.0041	0.03	59.5		Centromeric	TaeSat31-889	0.003	0.03	Centromeric	0.00	0.07
TtuSat25-320	320	0.0039	0.12	46.6	SF-6	Subtelomeric	TaeSat22-320	0.008	0.09	Subtelomeric	0.05	2.82
TtuSat26-732	732	0.0032	0.06	67.6		Multiple locations	TaeSat18-733	0.012	0.08	Multiple locations	0.03	1.83
TtuSat27-528	528	0.0025	0.02	56.6		Subtelomeric	TaeSat32-528	0.002	0.02	Subtelomeric	0.00	0.00
TtuSat28-175	175	0.0021	0.11	72.0		Subtelomeric	TaeSat34-175	0.0004	0.16	Multiple locations	0.04	2.61
TtuSat29-210	210	0.0020	0.05	60.0		Subtelomeric	TaeSat26-210	0.004	0.03	Subtelomeric	0.00	0.30
TtuSat30-543	543	0.0016	0.08	56.3		Centromeric	TaeSat28-543	0.004	0.11	Centromeric	0.03	1.64
TtuSat31-54	54	0.0001	0.21	72.2		Multiple locations	TaeSat33-54	0.0006	0.22	Multiple locations	0.00	0.00
		1.8905	0.11						0.12		0.03	1.96

Length (nt); abundance (% of genome); variation (%); A + T content (%); superfamilies (SF); FISH: scattered (satDNAs with scattered signal along the whole chromosome); (peri)centromeric (satDNAs with positive signal around the centromere of the chromosomes); terminal [satDNAs with signal localized in the terminal regions of the chromosomes (subtelomeres)]; multiple locations [satDNAs with undefined signal in a specific chromosomal region (terminal, centromeric, interstitial)] – see Table 3 for more information on FISH; *Triticum aestivum* homologous satellite to *Triticum turgidum* (Tae Homologous); the following columns [abundance (% of genome), variation (%) and FISH] are referenced to *T. aestivum* information. Divergence and consensus turnover rate (CTR); no sequences found (n.s.).

**Table 2.** Comparison of the number of base pairs and number of copies of satDNAs identified in durum wheat and bread wheat.

Satellite name	Length	Abundance (%)	No. of bp	No. of copies	Tae homologous	Length	Abundance (%)	No. of bp	No. of copies
TtuSat01-589	584	0.3919	47239626	80890	TaeSat01-584	584	0.4729	80175466	137287
TtuSat02-118	118	0.3329	40124637	340039	TaeSat02-118	118	0.3053	51760562	438649
TtuSat03-403	403	0.2037	24553998	60928	TaeSat06-403	403	0.1815	30771510	76356
TtuSat04-338	338	0.1670	20133816	59568	TaeSat04-337	337	0.2710	45945340	136336
TtuSat05-503	503	0.1651	19906283	39575	TaeSat05-500	500	0.2204	37366616	74733
TtuSat06-663	663	0.1263	15224202	22963	TaeSat08-663	663	0.1066	18072964	27259
TtuSat07-333	333	0.0764	9204339	27641	TaeSat09-335	335	0.1003	17004862	50761
TtuSat08-343	343	0.0716	8630664	25162	TaeSat07-343	343	0.1688	28618352	83435
TtuSat09-653	653	0.0633	7631491	11687	TaeSat19-653	653	0.0111	1881894	2882
TtuSat10-504	504	0.0583	7024053	13937	TaeSat11-506	506	0.0784	13291936	26269
TtuSat11-620	620	0.0273	3290742	5308	TaeSat15-620	620	0.0260	4408040	7110
TtuSat12-178	178	0.0255	3075770	17280	n.s.	n.s.	n.s.	n.s.	n.s.
TtuSat13-1463	1463	0.0240	2892984	1977	TaeSat14-1463	1463	0.0261	4424994	3025
TtuSat14-44	44	0.0217	2612034	59364	TaeSat13-44	44	0.0348	5899992	134091
TtuSat15-206	206	0.0215	2591610	12581	TaeSat10-206	206	0.0892	15122968	73412
TtuSat16-323	323	0.0213	2567502	7949	TaeSat17-323	323	0.0143	2424422	7506
TtuSat17-567	567	0.0196	2358633	4160	TaeSat16-567	567	0.0199	3373846	5950
TtuSat18-319	319	0.0138	1668018	5229	TaeSat23-319	319	0.0075	1271550	3986
TtuSat19-72	72	0.0116	1398962	19430	TaeSat27-72	72	0.0044	745976	10361
TtuSat20-1590	1590	0.0093	1122670	706	TaeSat21-1590	1590	0.0091	1542814	970
TtuSat21-318	318	0.0070	843780	2653	TaeSat25-318	318	0.0048	813792	2559
TtuSat22-322	322	0.0070	843780	2620	TaeSat20-322	322	0.0096	1627584	5055
TtuSat23-319	319	0.0051	609881	1912	TaeSat29-319	319	0.0035	593390	1860
TtuSat24-889	889	0.0041	495599	557	TaeSat31-889	889	0.0029	491666	553
TtuSat25-320	320	0.0039	470106	1469	TaeSat22-320	320	0.0079	1339366	4186
TtuSat26-732	732	0.0032	382511	523	TaeSat18-733	733	0.0121	2051434	2799
TtuSat27-528	528	0.0025	300061	568	TaeSat32-528	528	0.0019	322126	610
TtuSat28-175	175	0.0021	249406	1425	TaeSat34-175	175	0.0004	67816	388
TtuSat29-210	210	0.0020	241080	1148	TaeSat26-210	210	0.0045	762930	3633
TtuSat30-543	543	0.0016	192864	355	TaeSat28-543	543	0.0040	678160	1249
TtuSat31-54	54	0.0001	12054	223	TaeSat33-54	54	0.0006	101724	1884
n.s.	n.s.	n.s.	n.s.	n.s.	TaeSat03-2619	2619	0.2757	46742178	17847
n.s.	n.s.	n.s.	n.s.	n.s.	TaeSat12-369	369	0.0460	7798840	21135
n.s.	n.s.	n.s.	n.s.	n.s.	TaeSat24-338	338	0.0069	1169826	3461
n.s.	n.s.	n.s.	n.s.	n.s.	TaeSat30-1389	1389	0.0032	542528	391
		1.8905	227881102	829604			2.5316	429207464	1367987

The increase in the number of repeats in bread wheat compared to durum wheat may be due either to an increase in the number of original copies existing in the A and B subgenomes of durum or to the contribution of this same type of sequence by the D subgenome in bread wheat. In this context, it is interesting that the superfamily SF5 of *T. aestivum* is composed of two satDNA families, one is TaeSat16-567 which is found in all the centromeres of the B subgenome, and the other is TaeSat12-369 which is found terminally in most D chromosomes. Consequently, this last satDNA family has not been identified by RE2/TAREAN in the *T. turgidum* genome which lacks the D subgenome. Furthermore, homologous satDNAs to TaeSat03-2619, TaeSat24-338 and TaeSat30-1389 were undetected in *T. turgidum* (Table 2). However, a BLAST search of these satellites against the assembled genome of *T. turgidum* reveals scattered repeats of all of them in this genome (see below).

We have estimated the divergence between consensus sequences of homologous satellites shared by both wheat species (Table 1). The values obtained ranged from 0 to 16 % with a mean value of 3 % sequence divergence (Table 1). Correspondingly, the average rate of change (CTR) of these satellites is 1.96 × 10^−6^ substitutions per site per year, ranging between 0 and 9.38 × 10^−6^ (Table 1). It is striking that the intraspecific variation values of each satellite are always higher (in some cases reaching 25 %) than the divergence between species (Table 1).

### Satellite DNA location

**Fig. 2.**
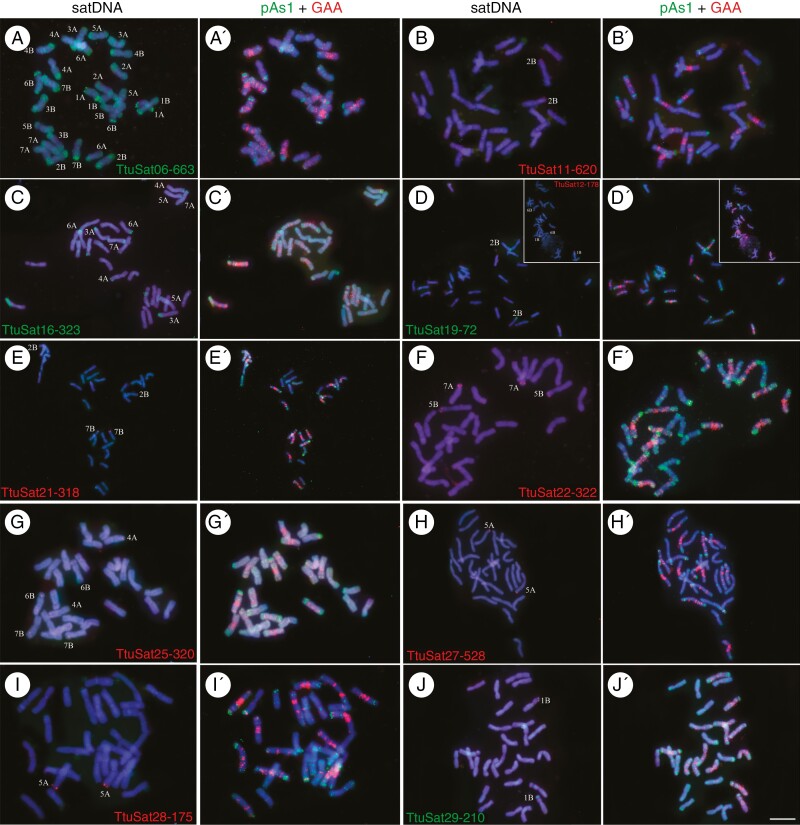
Cytogenetic visualization by fluorescence *in situ* hybridization of distal/subtelomeric satDNAs in metaphase chromosomes from *Triticum turgidum* cv. Capelli. DNA was counterstained with DAPI (blue). satDNAs were indistinctly labelled in red or green. Chromosome identification and orientation were confirmed by reprobing the chromosome spreads with the pAs1 (green) and GAA (red) probes (panels aʹ–jʹ). (a) TtuSat06-663, (b) TtuSat11-620, (c) TtuSat16-323, (d) TtuSat19-72 (insert TtuSat12-178), (e) TtuSat21-318, (f) TtuSat22-320, (g) TtuSat25-320, (h) TtuSat27-528, (i) TtuSat28-175 and (j) TtuSat29-210. Scale bars = 10 µm, except for panels d, e and h where the scale bar represents 5 µm.

**Fig. 3.**
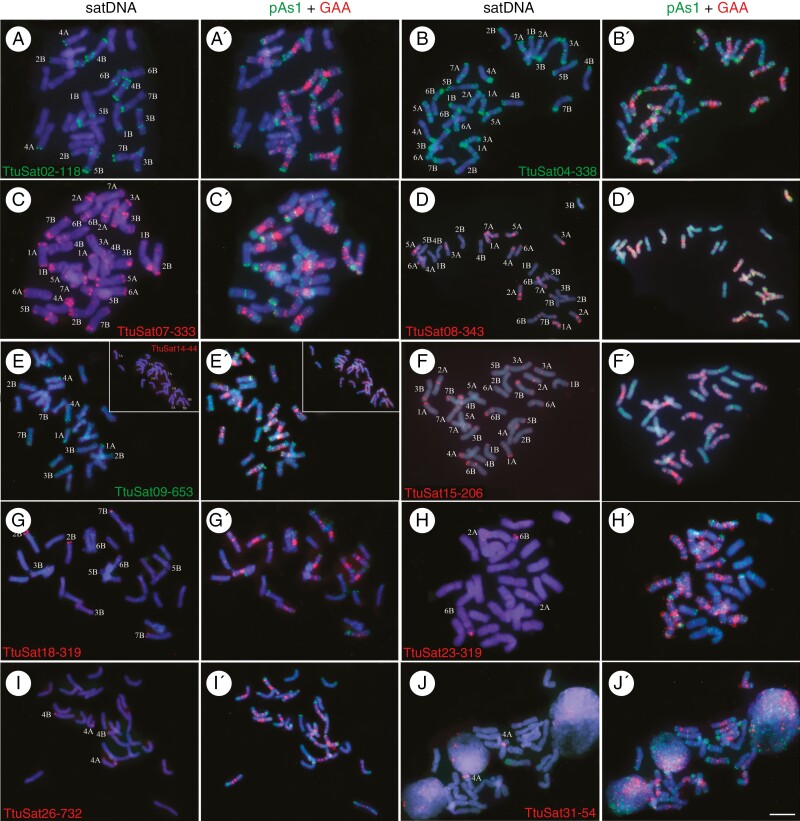
Cytogenetic visualization by FISH of satDNAs with a multiple location pattern (terminal/subtelomeric and some centromeric and/or interstitial signals) in metaphase chromosomes from *Triticum turgidum* cv. Capelli. DNA was counterstained with DAPI (blue). satDNAs were indistinctly labelled in red or green. Chromosome identification and orientation were confirmed by reprobing the chromosome spreads with the pAs1 (green) and GAA (red) probes (panels aʹ–jʹ). (a) TtuSat02-118, (b) TtuSat04-338, (c) TtuSat07-333, (d) TtuSat08-343, (e) TtuSat09-653 (insert TtuSat14-44), (f) TtuSat15-206, (g) TtuSat18-319, (h) TtuSat23-319, (i) TtuSat26-732 and (j) TtuSat31-54. Scale bar = 10 µm, except for panels d and f where the scale bar represents 5 µm.

**Fig. 4.**
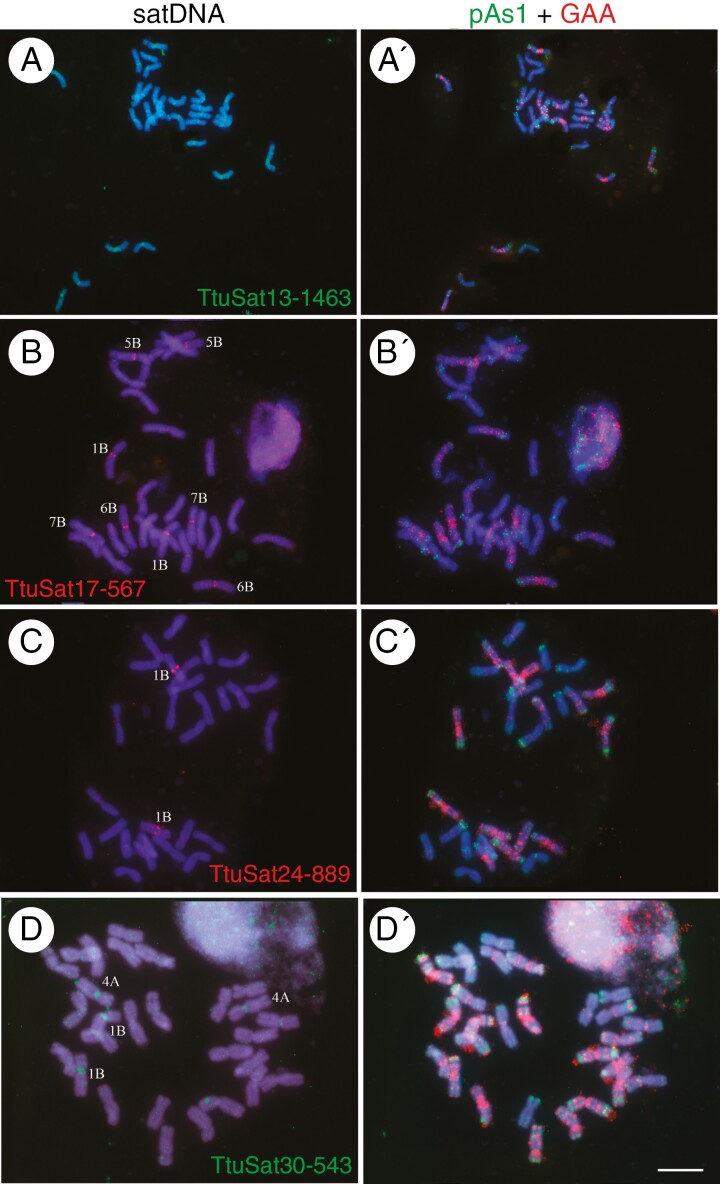
Cytogenetic visualization by FISH of centromeric satDNAs in metaphase chromosomes from *Triticum turgidum* cv. Capelli. DNA was counterstained with DAPI (blue). satDNAs were indistinctly labelled in red or green. Chromosome identification and orientation were confirmed by reprobing the chromosome spreads with the pAs1 (green) and GAA (red) probes (panels aʹ–dʹ). (a) TtuSat13-1463, (b) TtuSat17-567, (c) TtuSat24-889 and (d) TtuSat30-543. Scale bar = 10 µm except for panel a where the scale bar represents 5 µm.

**Fig. 5.**
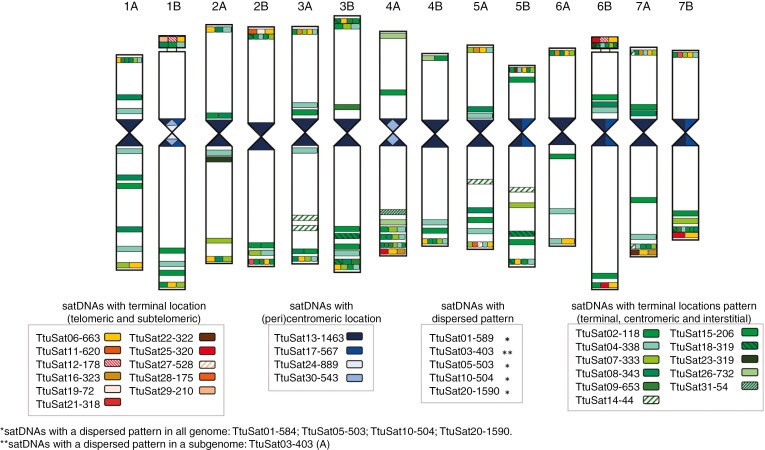
Idiogram of *Triticum turgidum* cv. Capelli chromosomes representing satellite DNA location identified in this work by fluorescence *in situ* hybridization (FISH). satDNAs were grouped according to their location patterns: satDNAs with distal chromosome location (telomeric and subtelomeric) in a brown colour scale; satDNAs with (peri)centromeric location in blue scale; satDNAs with multiple locations pattern (terminal, centromeric and interstitial) in green scale. *satDNA with a dispersed pattern in all genomes (TtuSat01-589, TtuSat05-503, TtuSat10-504 and TtuSat20-1590); **satellite DNAs with a dispersed pattern in the A subgenome (TtuSat03-403) are not represented on the ideogram.

**Fig. 6.**
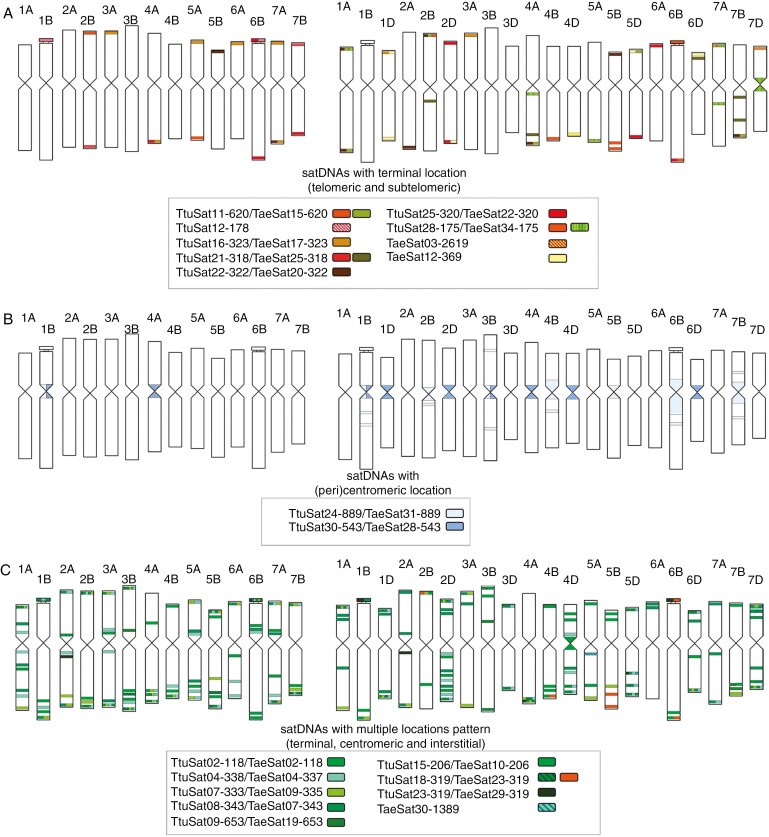
Comparative ideograms of *Triticum turgidum* and *Triticum aestivum* chromosomes representing satellite DNA with different locations between both species. satDNAs were grouped according to their location patterns: (a) satDNAs with distal chromosome location (telomeric and subtelomeric) in a brown colour scale; (b) satDNAs with (peri)centromeric location in blue scale; (c) satDNAs with multiple locations pattern (terminal, centromeric and interstitial) in green scale. satDNAs with a dispersed pattern are not represented on the ideogram.

Durum wheat satDNAs were organized into four different groups according to their FISH pattern (Figs 2–4; Table 3). The distribution patterns have been represented by ideograms in Fig. 5 and Supplementary Data Fig. S2 (independent ideograms according to the cytogenetic pattern). Eleven satellite DNAs displayed an exclusively subtelomeric pattern (Fig. 2). All of them, except TtuSat12-178, have a homologous satDNA in the CS bread wheat genome. TtuSat12-178 has a positive FISH signal in chromosomes 1B-organizer and 6B-organizer. Of the remaining, three show the same FISH pattern as their bread wheat counterparts (TtuSat19-72, TtuSat27-528 and TtuSat29-210) (Figs 5 and 6). However, in most cases (TtuSat06-663, TtuSat11-620, TtuSat16-323, TtuSat21-318, TtuSat22-322 and TtuSat28-175), more FISH loci are detected in bread wheat than in durum wheat (Figs 5 and 6). This is not only because these satellites are also present in some chromosomes of the D subgenome of *T. aestivum* (TaeSat08-663, TaeSat15-620, TaeSat17-323, TaeSat25-318, TaeSat20-322 and TaeSat34-175), but also because additional loci have been detected in their A and B subgenomes. Conversely, the satellite TtuSat25-320 in bread wheat has lost some of the conspicuous FISH loci visible in durum wheat (Figs 5 and 6).

**Table 3.** Summary of the fluorescence *in situ* hybridization (FISH) patterns for the different durum wheat (*Triticum turgidum* cv. Capelli) satellite DNA families identified in this work.

Satellite DNA	1A	1B	2A	2B	3A	3B	4A	4B	5A	5B	6A	6B	7A	7B
TtuSat01-589	D	D	D	D	D	D	D	D	D	D	D	D	D	D
TtuSat02-118		−/−/I + ST		ST/−/I + ST		ST/−/I + ST	−/−/ST	ST/−/I + ST		ST + I/−/−		−/−/I + ST		−/−/I + ST
TtuSat03-403	D		D		D		D		D		D		D	
TtuSat04-338	ST + I/−/I + I	ST/−/I	ST/−/I	ST/−/ST	ST + I/−/I + ST	ST/−/I + ST	−/−/I + I + ST	−/−/I + ST	ST + I/−/I + ST	ST/−/ST	−/−/I + ST	ST + I/−/	ST/−/I + ST	ST/−/ST
TtuSat05-503	D	D	D	D	D	D	D	D	D	D	D	D	D	D
TtuSat06-663	ST/−/ST	ST/−/ST	ST/−/ST	ST/−/ST	ST/−/ST	ST/−/ST	−/−/ST	−/−/ST	ST/−/ST	ST/−/ST	ST/−/ST	ST/−/ST	ST/−/ST	ST/−/ST
TtuSat07-333	ST/−/ST	−/−/ST	−/−/I + ST	ST/−/I	ST/−/ST	ST/−/ST	−/−/I + I + ST	−/−/ST	ST/−/ST	ST/−/I	ST/−/−	ST/−/−	ST/−/ST	ST/−/I
TtuSat08-343	ST + I/−/I+/I	ST/−/−	ST + I/−/ST		−/−/I + ST	−/−/ST	−/−/I + ST		I/−/I		ST/−/−	ST + I/−/−	ST + I/−/ST	−/−/ST
TtuSat09-653	ST/−/−			−/−/ST		ST + I/−/ST	−/−/ST							−/−/ST
TtuSat10-504	D	D	D	D	D	D	D	D	D	D	D	D	D	D
TtuSat11-620				ST/−/−										
TtuSat12-178		ST/−/−										ST/−/−		
TtuSat13-1463	−/C/−	−/C/−	−/C/−	−/C/−	−/C/−	−/C/−	−/C/−	−/C/−	−/C/−	−/C/−	−/C/−	−/C/−	−/C/−	−/C/−
TtuSat14-44					−/−/I + I				−/−/I	−/−/I			ST/−/ST	
TtuSat15-206	ST/−/I	ST/−/I	I/−/−	ST/−/I + ST	ST + I/−/ST	ST/−/I	I/−/I + I + ST	−/−/ST	−/−/I	ST/−/I + ST	ST/−/I	ST + I/−/−	I/−/I + ST	ST/−/ST
TtuSat16-323					ST/−/−		−/−/ST		ST/−/−		ST/−/−		ST/−/ST	
TtuSat17-567		−/C/−								−/C/−		−/C/−		−/C/−
TtuSat18-319				ST/−/−		ST/−/I + ST				−/−/I + ST		ST/−/−		−/−/ST
TtuSat19-72				ST/−/−										
TtuSat20-1590	D	D	D	D	D	D	D	D	D	D	D	D	D	D
TtuSat21-318				−/−/ST										ST/−/−
TtuSat22-322										ST/−/−			−/−/ST	
TtuSat23-319			−/−/I									ST/−/−		
TtuSat24-889		−/C/−												
TtuSat25-320							−/−/ST					ST/−/ST		−/−/ST
TtuSat26-732							ST/−/I	ST/−/−						
TtuSat27-528									−/−/ST					
TtuSat28-175									−/−/ST					
TtuSat29-210		ST/−/−												
TtuSat30-543		−/C/−					−/C/−							
TtuSat31-54							−/−/I							

The different lines show the result for individual satDNAs. Each column shows the information for individual chromosomes. The different subgenomes are differentiated by colour (A = blue; B = pink). Each cell represents: short arm/centromere/long arm; ST = subtelomere; I = interstitial; C = (peri)centromeric; D = dispersed.

Eleven satDNAs were identified in durum wheat mainly in the subtelomeric regions but displayed some additional signals in the (peri)centromeric and/or interstitial chromosome regions and the nucleolar organizers (Fig. 3). A homologous satDNA to all of them was identified in the bread wheat satellitome. TtuSat14-44, TtuSat26-732 and TtuSat31-54 satellites in *T. turgidum* share FISH patterns with their homologous counterparts in *T. aestivum*. However, we have found that the homologous satDNAs of TtuSat02-118 and TtuSat23-319 have more FISH signals in the A and B subgenomes of bread wheat in addition to those on some chromosomes of the D subgenome (Figs 5 and 6). Another case is satellite TtuSat31-54, which shows a single interstitial locus on chromosome 5A of *T. turgidum* while in addition to this locus, *T. aestivum* showed two subtelomeric loci on chromosomes 4 and 7 of the D subgenome. TtuSat08-343 and TtuSat09-653 satDNAs occupy a few more loci than those shared with bread wheat in the durum wheat genome, regardless of whether their homologues show any additional locus in the D subgenome of *T. aestivum*. The remaining satellites show particular FISH patterns that are of note. TtuSat04-338 is homologous to the repeat pAs1 sequence. Interestingly, this satellite has a different FISH pattern between bread wheat [mainly located in chromosomes of subgenome D and some chromosomes (1, 4 and 7) of subgenome A] and durum wheat (which show FISH loci in all its chromosomes). In addition, signals in the A chromosomes of *T. turgidum* are stronger than in those of *T. aestivum*. A similar scenario is found for TtuSat15-206 satDNA, although the size of the shared FISH signals (1A, 3B, 4A and 5A) is similar in both species. Comparison between durum wheat and bread wheat subgenomes A and B for satellite TtuSat07-333 also reveals a higher number of loci in durum wheat. Finally, TtuSat18-319 and its homologue (TaeSat23-319) share FISH signals on some chromosomes of the B subgenome although TaeSat23-319 only has terminal locations (Figs 5 and 6).

Four DNA satellites showed a (peri)centromeric hybridization pattern, all of them identified in the bread wheat genome (Fig. 4). TtuSat13-1463 and its homologue TaeSat14-1463 showed similar FISH patterns in both species, located in the (peri)centromeric regions of all A and B chromosomes. However, TaeSat14-1463 was also present in all D chromosomes in *T. aestivum*. TtuSat17-567 displayed strong signals at the centromeres of 1B, 5B, 6B and 7B, as its homologous counterpart in bread wheat and very weak signals in chromosomes 2B, 3B and 4B (equivalent to bread wheat too). TtuSat24-889 was (peri)centromeric (exclusive of 1B). In contrast, the homologue to this satellite was found (peri)centromerically in all B chromosomes. Finally, TtuSat30-543 was found only in pairs 1B and 4A while bread wheat showed (peri)centromeric signals in all chromosomes, being more intense in chromosomes 1B, 1D, 2D, 3B, 4A, 4D and 6D. See Figs 5 and 6 for comparisons between *T. turgidum* and *T. aestivum*.

We have identified five satDNAs that show a dispersed FISH pattern (see Supplementary Data Fig. S3). TtuSat01-589, TtuSat03-403, TtuSat05-503, TtuSat10-504 and TtuSat20-1590 showed a dispersed pattern throughout the durum wheat chromosomes. The same pattern but with some modifications was observed in bread wheat chromosomes (Gálvez-Galván *et al.*, 2024). We highlight TtuSat05-503 since its homologue in the bread wheat genome, TaeSat05-500, showed more intense signals in the chromosomes of the D subgenome than in the other subgenomes. It is noteworthy that although these satDNAs have a dispersed chromosome pattern, in some cases, stronger FISH signals can be observed in certain chromosome regions, possibly indicating a higher accumulation of repeats in those chromosome regions.

### BLAST search of satDNAs to the genomes of T. turgidum and T. aestivum

We performed a BLAST search of the genome assembly of *T. turgidum* (https://www.ncbi.nlm.nih.gov/datasets/genome/GCA_900231445.1/) with the durum wheat satDNAs (Supplementary Data Table S4). As we previously found for the satellitome of *T. aestivum*: (1) a considerable proportion of repeat units of most satDNAs analysed have been discarded in the assembly and many of the loci identified by FISH corresponded to regions where the assembly has collapsed into only a few tandem repeats or even directly generated a gap, and (ii) all satDNAs analysed have copies of short tandem arrays of the repeat unit dispersed throughout the genome in addition to the major loci detected by FISH. In this context, all satDNAs showing a dispersed FISH pattern returned hundreds/thousands of hits per chromosome composed by one, two or a few tandem repeats, coinciding with the scattered FISH pattern (Table S4), as we found for their homologous counterparts in the genome of bread wheat (Gálvez-Galván *et al.*, 2024).

Supplementary Data Table S4 also summarizes the BLAST search of four satDNAs from *T. aestivum* to the genome of *T. turgidum* that we could not isolate from the latter species (TaeSat03-2619, TaeSat12-369, TaeSat24-338 and TaeSat30-1389). BLAST searches revealed copies of all of them within the genome of durum wheat. Thus, there are five repeats (two complete and three incomplete) of TaeSat12-369 in chromosome 4A in the assembled genome of *T. turgidum*. In *T. aestivum* TaeSat03-2619 shows conspicuous FISH bands terminally only in chromosome 7D but many copies of repeats or partial repeats of this satDNA are also dispersed throughout the A, B and D subgenomes. Similarly, the search for this satellite in the durum wheat genome revealed fragments of this repeat distributed throughout all chromosomes (Table S4), which were undetected by RE2/TAREAN or FISH. TaeSat24-338 does not form visible FISH bands in *T. aestivum* but a multitude of copies of this sequence are distributed throughout its genome, forming short tandems at some loci as occur in *T. turgidum*. TaeSat30-1389 is observable by FISH at an interstitial/terminal locus in chromosome 5D and a punctate interstitial locus in 5A of bread wheat. In addition, there are some scattered repeats (often incomplete) on chromosomes 5B, 7A and 7D. It was not possible to isolate this satellite by RE2 in durum wheat. However, a search of this satDNA to the genome of *T. turgidum* reveals several of these repeats on chromosomes 5A, 7A and 5B (Table S4).

**Fig. 7.**
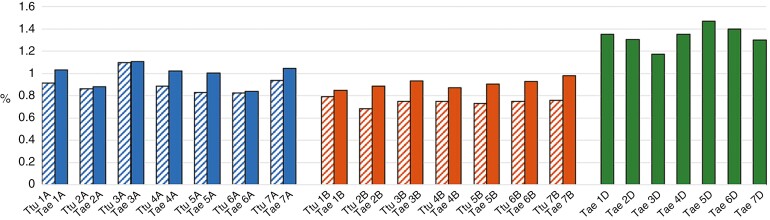
Summary of the composition of the repetitive fraction (satellite DNA) originating from individual clustering analysis in durum and bread wheat in each subgenome per species. The *y*-axis represents the total percentage of satDNA in the chromosomes of each species. On the *x*-axis, the different chromosomes (1–7) of each subgenome (AABB; AABBDD) are represented in different colours: blue (subgenome A); orange (subgenome B); green (subgenome D). A and B subgenomes corresponding to durum wheat are identified with striped bars. Abbreviations: *Triticum turgidum* (Ttu); *Triticum aestivum* (Tae).

**Fig. 8.**
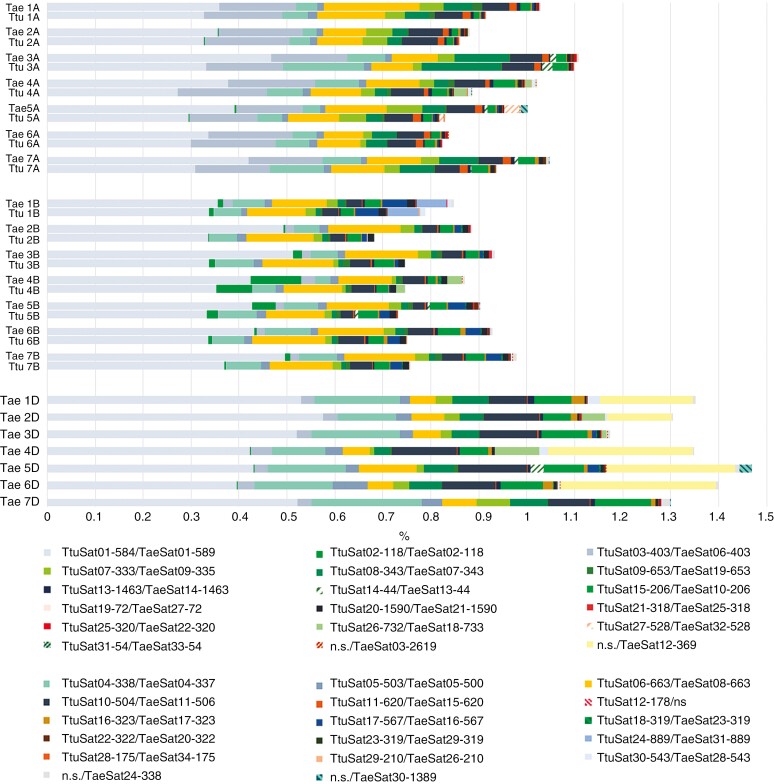
Summary of the composition of the repetitive fraction (satellite DNA) from the individual clustering analysis in durum and bread wheat of each satellite DNA of each species. The *y*-axis represents the different chromosomes (1–7) of each subgenome of each species. The *x*-axis represents the total percentage of each satDNA in the chromosomes of each species according to the chromosome size of the reference genome. The colour corresponding to each satDNA is indicated in the key. The colour used for each satellite is the same colour used to represent the satellite in the different ideograms. Abbreviations: *Triticum turgidum* (Ttu); *Triticum aestivum* (Tae); no sequences found (n.s.).

We compared the genome assemblies of both species to analyse the differences between them. Figures 7 and 8 represent data estimated in proportion to the chromosome size according to the reference genome of the species and show that each bread wheat chromosome has a higher satDNA content than its counterpart in durum wheat. Furthermore, it is noteworthy that the proportion of each chromosome of subgenome D that is satDNA is higher than that in subgenomes A or B. In addition, if we compare subgenomes A and B of durum wheat and bread wheat species, in general, subgenome A is richer in satDNA than subgenome B.

In general, the most abundant satDNAs in each chromosome are also the most abundant in each genome while the proportion of each satDNA per chromosome is slightly higher in *T. aestivum* than in *T. turgidum*. As displayed in Fig. 8, the highest percentage of satDNA corresponds to the same satDNA in both species. An important part of this percentage corresponds to the dispersed satDNAs in both species: TtuSat01-589/TaeSat01-584, TtuSat03-403/Taesat03-403 and TtuSat05-503/TaeSat05-500. Remarkably, TtuSat01-589/TaeSat01-584 are the most abundant satDNAs on all chromosomes of all subgenomes in both species, while TtuSat03-406/TaeSat06-403 are more abundant on the A subgenome in both species, in agreement with FISH results. TtuSat05-503/TaeSat05-500 have similar proportions in the A and B subgenomes in both species. However, the proportion in the D subgenome of bread wheat is higher (Fig. 8), as confirmed by FISH results (Gálvez-Galván *et al.*, 2024).

satDNAs mainly represented in the terminal regions (TtuSat02-118/TaeSat02-118, TtuSat04-338/TaeSat04-337, TtuSat06-663/TaeSat08-663, TtuSat07-333/TaeSat09-335 and TtuSat08-343/TaeSat07-343) represent a considerable percentage of the total satDNAs. Remarkably, TtuSat02-118/TaeSat02-118 are most represented on the B subgenome in both species, in agreement with FISH results. TtuSat04-338/TaeSat04-337 is represented in all subgenomes, highlighting the D subgenome of bread wheat (this satellite is homologous to pAs1as noted previously, a sequence used to specifically identify chromosomes of the D subgenome). TtuSat06-663/TaeSat08-663 are represented in similar proportions in the A and B subgenomes in both species, but are less abundant in the D subgenome of bread wheat. TtuSat07-333/TaeSat09-335 have similar proportions in the A and B subgenomes of both species, being more abundant in the A subgenome (see FISH results for both species). TtuSat08-343/TaeSat07-343 are more represented in the A subgenome of both species and also in the D subgenome in bread wheat.

Finally, we searched for satellites that were detected in this research as exclusive to either *T. turgidum* (TtuSat12-178) or *T. aestivum* (TaeSat03-2619, TaeSat12-369, TaeSat24-338 and TaeSat30-1389) in each of the accessions whose complete genome sequence is available from both species as well as species related to their parental genomes (Supplementary Data Table S5). For TaeSat03-2619, TaeSat12-369, TaeSat24-338 and TaeSat30-1389, hits were found not only in the CS cultivar (IWGSC CS RefSeq v2.1) and all bread wheat accessions but also in Svevo.v1 (the only *T. turgidum* complete genome assembly), as mentioned before, and in the diploid genomes [*T. urartu* (AA), *T. monococcum* (AA), *A. speltoides* (BB) and *A. tauschii* (DD)]. TtuSat12-178 was not found in the CS genome assembly (IWGSC CS RefSeq v2.1) nor five of the other 28 tracked assemblies, but several hits were variably detected (between three and 24715) in the assemblies of the remaining 23 accessions. In addition, the analysis revealed several hits in the genome assemblies of *A. speltoides* (BB), but not in the other diploid genomes.

Additionally, a Primer-BLAST search (https://www.ncbi.nlm.nih.gov/tools/primer-blast/) with ACTCACATATGGCCGGTTTT/AACACCTCGATAACTTGCTCA (the primer pair used for PCR amplification of the TtuSat12-178 satDNA; Supplementary Data Table S1) was consistent with those results but with one exception: the primer pair also aligned with various locations on different chromosomes of *A. tauschii* (DD) (Table S6).

## DISCUSSION

### What the analysis of wheat satellitomes contributes to the satDNA concept

Bread and durum wheat are two species of enormous agronomic and economic interest worldwide. Both are allopolyploid species with a huge genome whose major part (~85 %) is constituted by repetitive DNA sequences, mainly TEs (84.7 and 82.2 %, respectively) and satDNA (Maccaferri *et al.*, 2019; Feldman and Levy, 2023). In this paper, we analyse for the first time from a genomic perspective the complete set of satellite DNAs that compose both genomes (i.e. their satellitomes) comparing the results obtained here for the durum wheat satellitome with those obtained previously for the bread wheat satellitome (Gálvez-Galván *et al.*, 2024). Our results demonstrate that there are many more copies of tandemly repeated DNA in bread wheat (about half a million more) than in durum wheat (Table 2).

Study of the satellitomes of wheat species contributes significantly to increasing our understanding of how satDNAs are organized and how they evolve as well as our knowledge of the association between satDNAs and TEs (this paper; Gálvez-Galván *et al.*, 2024). Our results corroborate those of recent studies over the last 10 years (e.g. Pavlek *et al.*, 2015; Ruiz-Ruano *et al.*, 2016; Pita *et al.*, 2017; Vondrak *et al.*, 2020; Gržan *et al.*, 2023), which have revealed important findings that have changed our view of the satDNA concept and which have been clearly and correctly pointed out previously (Šatović-Vukšić and Plohl, 2023). Thus, in wheat species we found two types of arrangements for satDNAs: (1) some satellites occupy considerable pericentromeric, subtelomeric and interstitial heterochromatic regions, cytogenetically visible as conspicuous FISH signals (i.e. ‘classical’ satDNA loci), with additional short arrays or single repeat units scattered throughout the euchromatin; and (2) short arrays of satDNAs, disseminated throughout the genome without any relevant clustering (Garrido-Ramos, 2017, 2021; Šatović-Vukšić and Plohl, 2023). Furthermore, many satDNAs are related to TEs (Šatović-Vukšić and Plohl, 2023) and the involvement of TEs in the origin of satDNA has been suggested (Macas *et al.*, 2015; Meštrović *et al.*, 2015; Garrido-Ramos, 2017, 2021; Vondrak *et al.*, 2020; Jesionek *et al.*, 2021; Šatović-Vukšić and Plohl, 2023). In the case of wheat species, we found that 18 satDNAs showed homology with TEs, and not only those with a scattered pattern but also 12 out of those 18 satellites have a combined organization of ‘classical’ loci and dispersed short arrays (Gálvez-Galván *et al.*, 2024). Therefore, this relationship between satDNA and TEs could have had a major influence on the composition and evolution of the satellitomes of these species.

### The highly dynamic satellitomes of wheats

*Triticum aestivum* (bread wheat) is an allohexaploid species (2*n* = 6*x* = 42; BBAADD) that arose as the result of hybridization between the tetraploid cultivated emmer wheat (*T. turgidum*; 2*n* = 4*x* = 42; BBAA) and the wild species *A. tauschii* (2*n* = 2*x* = 14; genome DD) (McFadden and Sears, 1946; Dvorak *et al.*, 1998; Matsuoka, 2011; Brenchley *et al.*, 2012; Wang *et al.*, 2013). Domestication of wild emmer wheat occurred only ~10 000 years ago, while the origin of the bread wheat occurred ~8000 years ago and both *T. turgidum* and *T. aestivum* are cultivated species that have been propagated as inbred cultivars. Despite the very short time that has passed since *T. aestivum* arose, there have been important quantitative and qualitative changes between the satellitomes of the cultivars of both species analysed in this paper.

In this short period, the loss of a satDNA family, TtuSat12-178, in the bread wheat CS cultivar is remarkable. In the durum wheat genome it appears to form a locus detectable by FISH in chromosome pairs 1B and 6B, while in bread wheat these loci were not detected (Tables 1–3). In fact, a search for TtuSat12-178 in CS bread wheat revealed no traces of this satellite in its genome (Supplementary DataTable S5). This absence is also notorious in five other genome assemblages. However, there are 23 bread wheat accessions in which this sequence was found but with a highly variable number of BLAST hits. On the other hand, four satDNAs (TtuSat09-653, TtuSat18-319, TtuSat19-72 and TtuSat28-175) have reduced their repeat copy number in CS bread wheat (Table 2). Intriguingly, while TtuSat09-653, TtuSat18-319 and TtuSat19-72 have similar or greater numbers of FISH loci in the durum wheat genome than in the A and B bread wheat subgenomes (Figs 2–6), the homologue to TtuSat28-175, which takes up only one locus in durum wheat, takes up five loci in bread wheat but only one of them is contributed by the D subgenome.

How can we explain both the different organization of each of these five wheat satDNAs and the quantitative differences between the two wheat species? A birth–dissemination–clustering process has been proposed to explain the origin of satDNAs (Ruiz-Ruano *et al.*, 2016). It is accepted that tandemly repetitive DNA arises through molecular mechanisms of unequal exchange. For example, unequal crossing-over may duplicate a sequence generating a short array of a repetitive DNA sequence which we could consider the ‘seed’ of a future ‘classical’ satDNA locus visible as a conspicuous FISH block (Smith, 1976; Dover, 1982; Ruiz-Ruano *et al.*, 2016). Transposition or the re-insertion of replicative extrachromosomal sequences may be responsible for the dissemination of such ‘seeds’ throughout the genome (Dover, 1982; Ruiz-Ruano *et al.*, 2016). These ‘seeds’ can be amplified by generating longer arrays at different locations (‘classical’ satDNA loci), often located in all chromosomes of the genome, but also as a single locus in one or a few chromosomes (even if there may remain multiple ‘seed’ loci scattered in other parts of the genome). Furthermore, from an ancient ‘library’ of multiple satDNA families, each satellite may be amplified differently in different descendant species, which results in species-specific satellitome profiles (the library hypothesis; Fry and Salser, 1977). These mechanisms may in turn be responsible for the decrease in the number of copies of a tandem array, or even for its disappearance. Thus, for example, unequal crossing-over is a molecular mechanism that generates two recombination products, one in which the tandem is lengthened and the other in which the number of copies of the tandem is reduced (Smith, 1976), depending on the random fixation in the population of one or the other product (Camacho *et al.*, 2022). This chance event could explain the reduction of the number of copies of several satellites; or the disappearance of satellite TtuSat12-178 in CS bread wheat, and some other bread wheat varieties, but not in all bread wheat accessions. We must also consider that, as we have seen in the assembly of the durum wheat genome now and earlier in the assembly of the bread wheat genome (https://www.ncbi.nlm.nih.gov/datasets/genome/GCF_018294505.1/), the genomic assemblies available to us are not complete in terms of repeated sequences. This could explain the absence of TtuSat12-178 in the CS assembled genome and the large variation we observed for this satellite in the sequence assemblies of the other accessions. However, neither the satDNA mining performed by us nor the PCR left any evidence of this satellite in the genome, so it is assumed that this satellite is not present in the genome of the CS accession. The quantitative changes described for these satDNA families might have been accelerated by genomic changes that would have accompanied the hybridization/polyploidization events involved in its origin, especially those derived from the intensive selection processes accompanying domestication. In fact, breeding and artificial selection have led to significant genetic changes in the genomes of durum wheat and bread wheat (Maccaferri *et al.*, 2019; Levy and Feldman, 2022) and domestication in maize has also led to important changes in satDNA content (Bilinski *et al.*, 2015). Thus, breeding and artificial selection would explain the important differences for TtuSat12-178 amounts among the different bread wheat lines (Supplementary Data Table S5). On the other hand, the curious case of TtuSat28-175 satDNA and its homologous counterpart in bread wheat would imply alternatively either the expansion by transposition to new loci after hybridization/polyploidization/domestication from the locus taken up by this satellite on chromosome 5A of the donor durum wheat genome or the generation of new conspicuous loci visible by FISH from previously existing ‘seeds’ at those locations. By analysing the genome of both species, we have found that those ‘seeds’ already existed in the durum wheat genome on chromosomes 1A, 4A and 7A where new loci have appeared in bread wheat, which supports the second hypothesis. Interestingly, the number of sequences in this family has decreased in the transition from durum to bread wheat, which implies a reduction in the number of copies of chromosome 5A and an increase in the number of copies in the other loci (not large, but enough to be revealed as visible loci by FISH).

On the opposite side, copy number expansions in the bread wheat genome have occurred during this time for 21 durum wheat satellites (Table 2): TtuSat01-589, TtuSat02-118, TtuSat03-403, TtuSat04-338, TtuSat05-503, TtuSat06-663, TtuSat07-333, TtuSat08-343, TtuSat10-504, TtuSat11-620, TtuSat13-1463, TtuSat14-44, TtuSat15-206, TtuSat17-567, TtuSat20-1590, TtuSat22-322, TtuSat25-320, TtuSat26-732, TtuSat29-210, TtuSat30-543 and TtuSat31-54. In some cases, this increase in the number of satDNA repeats has been aided by the contribution of repeated sequences by the donor diploid parent of the D subgenome (*A. tauschii*). Although TtuSat04-338, TtuSat07-333 and TtuSat15-206 have more loci in durum wheat than in bread wheat subgenomes A and B, the number of copies in the latter is higher due to the contribution of new loci coming from subgenome D. In many other cases, the evolutionary dynamics of satDNA itself has led to the increase of repeat copy number by increasing the number of loci within the A and B subgenomes. This may be either by transposition from pre-existing loci in the tetraploid donor genome (*T. turgidum*) or by amplification of loci present in the A and B subgenomes as ‘seeds’ not detectable by FISH in durum wheat as discussed above. Curiously, five satDNAs (TtuSat16-323, TtuSat21-318, TtuSat23-319, TtuSat24-889 and TtuSat27-528) have maintained a similar repeat copy number in both species despite an increase or decrease in the number of loci visible by FISH in bread wheat, expanding on the idea of high dynamism of wheat satellites.

Conversely, four satellites identified in the bread wheat genome have not been isolated in the durum wheat genome: TaeSat03-2619, TaeSat12-369 (which is part of the SF-5 superfamily), TaeSat24-338 and TaeSat30-1389 (Gálvez-Galván *et al.*, 2024). The first two satDNAs showed bands visible by FISH only in the bread wheat D subgenome (Gálvez-Galván *et al.*, 2024) which may in part explain the difference. In addition, both species (*T. aestivum* and *T. turgidum*) also contain repeats of these four satellites scattered in some chromosomes of the A and B subgenomes (Supplementary Data Tables S3 and S4). It is enlightening then that the ‘germ’ of these satellites may have been present in the ancestral genomes of wheat species and that, according to the library hypothesis (Fry and Salser, 1977), two of them (TaeSat03-2619 and TaeSat12-369) were amplified, generating loci visible by FISH, in the donor species of the D subgenome of *T. aestivum* and only traces of these sequences remained in the A and B subgenomes of both *T. aestivum* and *T. turgidum*. In fact, BLAST hits of all these four satDNAs were recovered in all diploid genomes analysed (Table S5). Interestingly, there are some variations in copy number of each of these satellites among the different bread wheat lines analysed (Table S5) which supports the role of selection in changes among accessions.

Most of the satellites analysed have undergone a high rate of evolution (Table 1). In a very short time, significant sequence divergence has occurred between the homologous satellites of both species, accompanied by high intraspecific diversity. High intraspecific diversity can be explained by the evolution of these species, given that they are of hybrid origin, in addition to the great dynamism of the satellites as we have just discussed. Despite this and the short time after the occurrence of *T. aestivum* (~8000 years), the average divergence between the consensus representative sequence of each species is considerable, which translates into an average high rate of change, two orders of magnitude greater than any known satellite (e.g. Navajas-Pérez *et al.*, 2009; Camacho *et al.*, 2022). On the other hand, the rate of evolution largely differs between satellites, confirming our previous suggestion (Gálvez-Galván *et al.*, 2024).

Taking all these data together, we conclude that wheat satellitomes are highly dynamic, showing an accelerated evolutionary rate. This high dynamism might be possibly influenced by genomic changes that would have accompanied the hybridization/polyploidization events involved in their origin, highlighting the involvement of TEs in the origin of new satellites and their dissemination, and in particular by those exerted by the intense selection processes that have accompanied domestication. Thus, in a very short evolutionary time relevant quantitative and qualitative changes have occurred in the satDNAs of durum wheat and bread wheat cultivars, which contrasts with other satDNAs (e.g. Garrido-Ramos, 2017, 2021; Šatović-Vukšić and Plohl, 2023).

### The polymorphic nature of wheat centromeres and subtelomeres

Both durum and bread wheat species share the same four centromeric satDNAs. However, all of them have increased loci and repeat copy numbers in the bread wheat genome. Thus, TtuSat13-1463 and its homologue TtaSat14-1463 in bread wheat are present in every chromosome. This points to a relevant role of this satellite in centromeric function because it would be conserved in three species (bread wheat and its two parental species, durum wheat and the diploid donor species). This satellite has homology with transposons (Supplementary Data Table S3). The nature of centromeric function has long been debated and it has been accepted that it is epigenetically controlled (Wang *et al.*, 2009). Thus, although satDNAs should support a structural role for the functional basis of the centromere (Hartley and O’Neill, 2019), there is a total absence of centromeric satDNA sequence conservation between species (Garrido-Ramos, 2017, 2021). In this context, it is particularly relevant that, in the absence of conservation, any satDNA sequence that meets certain structural requirements can play this role (Kasinathan and Henikoff, 2018). It is also particularly relevant that this structural role can also be played by diverse types of TEs, as demonstrated in plants in particular (reviewed in Garrido-Ramos, 2021) and especially in wheat species (Liu *et al.*, 2008; Su *et al.*, 2019). In this context, it is also plausible that different repetitive sequences can replace others in the centromeric region of different species, even if these are phylogenetically closely related (Ávila Robledillo *et al.*, 2020; Camacho *et al.*, 2022). Therefore, it is especially relevant that even though only 8000 years separate durum wheat and bread wheat, expansions of repeat copy number and the number of loci of the other three centromeric satellites are occurring in bread wheat, something that may eventually mark differentiation between subgenomes (TtuSat17-567 and TtuSat24-889 are specific to all the chromosomes of the B subgenome in bread wheat while these satellites are only represented in one or a few chromosomes of the B subgenome in durum wheat). Such differentiation may be crucial for the correct identification and match of chromosomes during meiosis (Calderón *et al.*, 2014; Aguilar and Prieto, 2020; Gálvez-Galván *et al.*, 2024) and would be consistent with the observation that wheat centromeric satellites are divergent between subgenomes (Su *et al.*, 2019). Dynamic changes inserting differentiated centromeric retrotransposons can also contribute to the subgenome differentiation in polyploid wheat species (Li *et al.*, 2008, 2013). Interestingly, TtuSat30-543 has expanded from just a pair of chromosomes in durum wheat to all chromosomes in the bread wheat genome, although in this case there were important quantitative differences between chromosomes (Gálvez-Galván *et al.*, 2024). Taking all these data together, we can conclude a high level of dynamism also in the evolution of centromeric satellites and that important changes are occurring throughout the evolution of wheat species at the centromeric level.

The subtelomeric regions in wheat are highly polymorphic (Navajas-Pérez *et al.*, 2009; Hartley and O’Neill, 2019). Despite the complexity of the tetraploid genome of durum wheat, as with hexaploid bread wheat, both behave as diploid species during meiosis. During this process, homologous chromosomes (equivalent chromosomes of the same subgenome) must recognize and associate for recombination and proper gamete segregation. This homologous pairing is particularly efficient in polyploid species and is genetically controlled (Zhang *et al.*, 2014). The mechanisms and proteins involved in the correct matching between homologous chromosomes during meiosis are still an enigma. During prophase I (zygotene stage), wheat telomeres approach each other forming the bouquet structure that probably facilitates homologous chromosome coupling, bringing the terminal regions of the chromosomes closer together. The telomeric sequence is nearly the same in all chromosome arms, so these polymorphisms in the subtelomeres could contribute to the specificity of the correct homologous chromosome identification at the beginning of meiosis in wheat (Aguilar and Prieto, 2020, 2021; Serrano-León *et al.*, 2023). The terminal regions of durum and bread wheat chromosomes are rich in repetitive elements (satDNAs and TEs) and we have proposed that satDNAs contribute to the polymorphism that exists in the terminal region of the chromosomes and this polymorphism may be crucial in correct homologous pairing (Gálvez-Galván *et al.*, 2024). In both wheat species, some satDNAs are exclusive of one chromosome arm (TtuSat11-620, TtuSat19-72, TtuSat27-528, TtuSat28-175 and TtuSat29-210 in durum wheat) which might simplify sequence specificity in these chromosome arms, and the differential distribution of satDNAs and high variability among homoeologous chromosomes might also contribute to that specificity. The expansions and contractions of repeat copy number and of the number of loci of subtelomeric satellites in both species may eventually mark differentiation between subgenomes and between species.

## SUPPLEMENTARY DATA

Supplementary data are available at *Annals of Botany* online and consist of the following.

Figure S1: Sequence in fasta format for each satDNA family. Figure S2: Ideograms of chromosomes of durum wheat (*T. turgidum* cv. Capelli) representing satellite DNA location identified in this work by fluorescence *in situ* hybridization (FISH). Figure S3: Cytogenetic visualization by FISH of satDNAs with a non-banded pattern (dispersed) in metaphase chromosomes from *Triticum turgidum* cv. Capelli. Table S1: Primers designed in this study to amplify each satDNA family. Table S2: satDNA families previously published in other studies. Table S3: Homology to satDNAs with transposable elements. Table S4: BLAST results of each satDNAs to the *T. turgidum* genome. Table S5: Search for the satellites that we detected in this research. Table S6: Primer BLAST results for satDNA TtuSat12-178 in the different *T. aestivum*, *T. turgidum*, *T. urartu*, *T. monococcum*, *A. speltoides* and *A. tauschii* assembly genomes public on NCBI webpage.

mcae132_suppl_Supplementary_Figure_S1

mcae132_suppl_Supplementary_Figure_S2

mcae132_suppl_Supplementary_Figure_S3

mcae132_suppl_Supplementary_Table_S1

mcae132_suppl_Supplementary_Table_S2

mcae132_suppl_Supplementary_Table_S3

mcae132_suppl_Supplementary_Table_S4

mcae132_suppl_Supplementary_Table_S5

mcae132_suppl_Supplementary_Table_S6
