# Supplementary material for: The highly dynamic satellitomes of cultivated wheat species
Source: Ann Bot. 2024 Aug 30;134(6):975–92. doi: 10.1093/aob/mcae132 (PMC11687632; doi:10.1093/aob/mcae132)
Supplement: mcae132_suppl_Supplementary_Figure_S1 [file mcae132_suppl_supplementary_figure_s1.docx]

Figure S1. Sequence in fasta format for each satDNA family.

>TtuSat01-589

AATAAGGAGGCACTTCCTTGCGGCCGCCGTGGACCCAGCTGTCAGCCTCTCCACGTACAGTACTCTTCCGATGGAAGTCGTTCGTTGACCACGTTGACCACGCCGCGCCGAGAGCACCAGGGCGGTGGTCTGGACGACGGCGAGGCCTAGGAAGGGGACGACGCGGAGCCGGGGAAGACGCAGCAGTGGAAGCCCGCGCGGAGAGGAGTACGAGGGTTCACTGGTTCGGCTGCGGTGTGAGGCTGCCGTCGCCGCAGAATAACAGGGGGTGTGGGTGAGTAGAGGGATGGCCTGGCCAGCGGTGGGAGTAGTAGGGGGCGGTGAGGCCTCCGCGGCATCACAGCCGGCCACGGGAGGCAGGAGCACGCGGCACGACCGGCGCTGCTTTGGGCGGCTGGAGCAAGAAGACCAGAGGTTGAAGAAGCACTACGGCCGTTGGATGGACATCGTACGGTCACTGGAGCTAGAATCGTTCATATTGACTAAGTTGACAAAGCCCTCCGTCCCCGTCAACTTAGTAGGCCCACAAGTCAGCCTGCCACTATACTGGGTCCCAGCTAACAGGGGGAGTATTCATTTTTTTGTGCGT

>TtuSat02-118

AACGCACCTTGGGGTTCGTCAATCATGGAAATCACTCCGGGACCCCAAAACAGTGAGTAATAGTCCATGAAACGGGCCAGAATCGGCCAAAACTGCGAGTGTTGATGACCGACACGTA

>TtuSat03-403

CTTAATATCTGTTTTGCCATGTTCCAGTTTTCTACTAAGTCTGAATCTGTTAACGATAATTGCTATGTTCACATGGTTGCCATTGTAGTGATCCGTGCCTCTTTTGAGCATGATCAGTAAGGATGTTTTGTAGATATTGTTGTGCTCTATCCATCCATGTCTTTGTTTGCAATTATGGAGCACCCTAGCTTGACTCAATCGAGCTCTACTTTTGCTATAAAATGTTCCTGGCAGAATGTTTACATGTTAAGCATTTTTGCCGAGGTTGTTGTAGTTGATCCATGCATGCTATGATGTTGTTCTTGCCATGTTTAGCTTCTATGCCATGTCTACTTGCTGGGTGTATGCTTAGTTTGTCATGCAATGCCTTGTGGTGAGTGCATCGAGCTCGTAAACATGCCTA

>TtuSat04-338

GAGTAGAGAGGGTTACGGCAAAAACTGGATGCACTTCGTGTACAAACTGGACAATCTCTTTCGAAGTATCAGGGTTTCGGACGAAAACTCATCTGTTACAAAGGGATTTCATTTTTTTGAACTTATTTGAACTCCATAGTTTTTCTGTGTTCAAAATGCACCATTCAAAGCCACATCATCAATTTTCAACCCTTTCTGACATCATTTGTTATTTTTCATGCATTTACTGATTATTTTGAGCTATAAGACCGTGAAATTGAAAATCACTACAAAATGAACTCTGAAAAGGTTGAAAGTTGGCATGGTATCATCATTTCACCCACATAGCATGTGCGAAA

>TtuSat05-503

CGTAATCACTGCCTCTTTTCGCCTTGTATTTTCTTCTACGGGCAACATGGAACGACATGATATCCATGCTGAAATTTAAACTTATTCAGGGTTCGTTTGGCCTTTTTATACACTAATTGAGTTTCCTAGGCATTTAATGTCCATAATTCAAATCTGAACTACAAATACATGCTCCAGTTCACCAAAATGGCTAGAAAAATTATACATGTGTCCTTGGGTGCATGTTTAGGTCCCATGCCAGGAATGGGAACGAATTACAACCGTACCGGTGTCCTGGCTCGTCCTCAAACATTTGGAATCTCGGTTTTTAAATCCCCATAAATCCAAAACTCACCAGAAAGTCATCAAAGGTGGCATGGTGTCACGTCATGGCACATATATGTCGTGGTAAAAACATTGTCCAATTTGGGCCAAGCTTTATTACAAACCTCTTACAAACCGGAGCTTCTCTCAAAGAAGCCTCGTGGTTCCGATAGGGAAACGTGTTCCCCTTGTGGGCGAAA

>TtuSat06-663

TCTTGGAATTTTTTTGGTGTTTCTCTGATTAAATAGATACTTATGTACCTAGAAATGATTTTTGGAAAAAATAAAGAGCAAACTATGAGGCAGCTGCAGTTCAAATTTGACCCGCTTCCTACTGAATCGGCGGGAATTTGTCTTTTTCACGAGAGGTGATGAAAACTTTTTACAACCCAACCATTTTGTCAATTGTGCATTAAATATGTCCTAGTATTTTAGAAAAATGATTTGGTCCAATTTTGCAACAATTATTTGGTAGGTCCTTCACAAAAAAACCTCCTTTTGGGCACTCGAAAAATGGAAAATGGTTTTTTCGTCCAAAGAAAATGAAAACTTCCTTAGGCAACATTGTTTGCCATTCCAATATGCACCCTTGTGCACAATATGAGATCATTTGAACAAACTATGCCATGAATGTGGCCATAAGATTGATCATTTGGCTTGAAAGCCATGAATCTTCACGCATGATAGCTCATTTCTGAGAACACTTTTTTAAAATAATTGCCGTATTACAAGTTTATTATTTTTCCTGGAAACTTGGTCACATATAATGACACAATGCGAAGGTTTTCCAATTTTTTGATTTTTTTTGAATTTTTTATGCCCGTTTCAAAATGCGGTCAAAACGGCGGGAATGACCGTTCCTAGCTAGTGGTTGAA

>TtuSat07-333

GGCGCCGCCGCCGGCACCTGGAGGGGGGAAACGGACGCGTCGGGTCTACACGGAGTGGATATTGCTGTGGGTTTGGCCCGGATGTTGCTCCGAGGTGTTCCTATGGGCTGACCTAACACAACCGGGTGGAGAGGTCCACTGGTCAAAGCCCGTCCGTGCAAAGTCAAAGGGCTAGATCCCGTGGTCAACCGCTACAGGGTTAGCCGGGACGGGGGCCCTGGGGGGCTAGCATGCCACCGGAGCGTCGGACCGGGCCCTCATACATGCGGGGTGGTGTTGTGGCATGTCCGAAGAGGCGGCACGCGTCTCCGGGGTGTGGCCCCCCTCACGGAC

>TtuSat08-343

GTTCATTTCATAGTGATTTTCAATTTCACGGTCATTTAGCTCTCTAAAACAATTAAGTGAATGACCGAAAAACAACGCATGATGTCAGAACATGTTGGAAATTGATGACGTCGCTTTGAATGCTGCATACTGAACACAAAAGAAGTCCGGAGTTCAAATAAGTTTAAAAAACATTGAAGTGCCCGTGTAACAGATGAGTTCTCGTCCGAAACCCTGATACTCCGAAAGAGTTTGTCCAGTTTGTACACGAAGTGCGTCCAGTTTTTGCCGTGACCCTCTCTACTCTTTCGCACATGCTATGCGGGTGAAATGATGATACCATGCCAAGTTTCAACATTTTCAGA

>TtuSat09-653

TTTTTCATTTTTTGAATGCTAAAGACATGCGTTTTTCGTGAAGCGGCTACAAGGAGGGTCACCCCAAACGGCGCCATTCCATGTCTATCTCAAAGTAGACCCTATTTTACGGACGGTCGCCAAAAAGCATGCATTTCCGACCCTCGTAGCTACTCCCGGCAATTCAGACCACCTTCGGCCGATTCAGCTGGAACCGGCTGGAATTTGAACTGCGGGTCCTCCATAGCTTGCCCGTTATTTTCACTAAAAATGCTTTTTAGCTTCACAGGTAGGCATTTCATCACAGAATCGACAACAGATTTGCACGGCTCAAACCCTAGCCACGGACGGCCGCCGCGACGAAATCGGCCGGTTTTTGAAAACACCGTAAAAAATTCAAAAAAATGGGAGACCTCCGCGTCACATCATCAAATGTGGCCTACCAACTAGCAAAAATACTAAACTTGCAATACCGGCGTTTTCTTGAAAAAGTGTTCTCAAAAACGACCTACCATGAACGAAGATTCATGGCTTTCAAGCCAAACTAGCAATGATATGGCCGCATCCGTTGAATAGTTTCTGATAATATGCCCAAATTTGGCGCATGCCTCCGTCTTGTGATGGCAAACAATGTTGCCAAAGCGAGGTTCCAACTTGTTTAACAAGAAAAACCG

>TtuSat10-504

ATTAAAAAATGATCAGAAAAACATGAAACCTTGCTTGATGTAATGTCATGCCACCAAGATGATGTGGTAAAAAAATTGGCATGTTTGACGAAAGTTTGGACACACACCCCTCACAAACCGGAGCAACTCACTAGAAGGCTCGTGGTTCCGAGAGGGAACAATGCATGTTTGATGACGAACGGGAGATAGCTTCCTCTTACGGCCTTCAAATTTTTTCTACGTTTAACGTGCACTACTACAACTGTCATGTGAAAATTTGGAAAATTTCAGGGGTCATTTGACCTTTTAAAGACATTTAAGTGATTTTCTAGCCATTTAATGACCGTAATTCAAATTTGAACTACATCTACATGCAACGGCTAACCATAACGGTTTGAAAAATCATATTTGTGTACTTGTGTGCGAGTTAATTCCATGTGCAGTAAATTAGAAGGAATTTTCAAACATATTGGTCTCACGGCATGGACACATGCATGGAGTGCCATGGCATTTTAATTCCAAAAA

>TtuSat11-620

ATAATTTGGGCATATGAAGGAACTGTATAAATTTCATGTCATTTAGAGATATAAAAAAGGTACTTCCTTCACAATGCTTCTAGGTGGACAAAAACTTTGGAAATTTGCCGAGGAAGATTTGCTAGGCAAATGGAGCTGAATTTTGTCATGCGGTAATGATTTGGATAGGAAAGAGTGCCCAAAAATTCCGAGGGCAATCAAGAATATATAAATAGCACTTCCTTCATAAAGTGTTGTTATGGACAGAAACTTGGGAAAACTAGTGAGAGAGATTGGATGAATGAAATGAGCTCAAAATTGGTGTAGGTAAGTTACATAGGTATGGTCATGCGCTGGTAAATTTTCAGATCATTTGGGTAAGCCTAGCTAGTACTTACTTCACAAAGCTTCTCTCGAGGTAGAAACTTTGGAAATTTCCCGAGAAAGATTTACTAGGAAAATTGAGCTGAATATTATCATGTGGCAATGATTTGGGTATGGAAGAGTGCCCGAAAAGTTTGAGGGTAATAGGAGGGGTCTATATAACACTTGCTTTGCAACGTGCCAATTTGGCCATAAAATATAAATTGAACCTGGGCTCACATAGATGATTTGACTGAGCTGCAATTTGGAGGAGGGTG

>TtuSat12-178

ACTCACATATGGCCGGTTTTAGGCCAAATTCACAACATGAGGTCATATATCGGTACCTAATTTGGCAAAATGTTGTGAACGGCGCTTGTCGAGGATGGTTTGGATAGCAAAAAGTTCTTCTGGCCGTGCCATAGATGAGCAAGTTATCGAGGTGTTTTTGGCCGACCTTTGTTTTTCG

>TtuSat13-1463

GCAAGTGAGCATGTGTATAATTTAGTGTGTGATTTGAAGATCAATACACATGGCAATATGCCAAAAAAAGTGCCCACCTACCAGAGCAGGAGAATTCATACTCCACGGCCTGCATACGACTAGGAACCCTGCGGTGCATGGGCCAGGATGCAGGCCCGTGGTGCAGAATATCTCTGCTGCTAGTAGGCCCCACGGGGCAGAGGGGACGAGAGTTAGGCAATGTACTGCCTGCAATTGATAGGAGCTTGTGAGGGGAGTCGCAGCCGGGTATATAAACCGGTGCGGCGCTCTCTCGCTTGGCGAGGTGGGACTAAACTCCCACCACCCAACGGCTATGCGCTGCGATGTGCCCTGCGCGGCTCCGCCTTGGGCCCAATTCGGGCGGGCTGGCCCAGGGCAGCCTTACTCCGCTCGCTGATACGTTTTAAATTTTCTTTGTTTCTTTTCTTGTTTATATTTCTAATATTTAAAATCAAATCTCTTTTTTCTATATTATTTCTAACAGTGTTTACTAAAATTTAAAAAATATGCCAAATTCTGTTGTGTTTTATTTCATTTGTTCGTTCTCTTTTGTATATTGATAATATTTACGAAATATAAACTAAAATGGTGTGTTTTATTTCAATTCTTCATTCTAATATTTCTTATCTTTATTATTTTTGAAATATTTTTAGTTTGTAAGATAAAATGTTCTGTTTTATTTCACTTCTTCATTCTAATATTTGTTCTCTTCTTTATATTTGAAATATTTCCAAAATGTAAGATAATATGTTATGTTCTATTGCACTTCTTCATTATTTCAATTCTTCATTCCAAATTATAAATTTTATTTTCTGTTTGATACTTGAAATATTATTAAATCAAATCTAGTTGGGACAGTCGCATGCATACCTCCACATGCACCATGTACTAGAAGCAGGTTTACCTTCCCGGTCTCACCAATTCAGACCAAACTGTATGCATACCCAAGGATGATCATGGCAAGCATGCATGACAAGTATCGTTCATTTCCGACACTCGATGCATTTTCTAGGGTTTTTCCGGCGACAAAACCCTACAACACTGCCCCCACGTGACGCAACGTGCGTTTTGTGTCCGAATTCGTGCACACCTTGCCTGGGGGACCACATTGGCCATGCCCACATGGTCCCAAAAGTTGGGGTCATCTCATGCATGCCTCCACATGCACCATGTACAAGCAGGACCATTCGGGTCTGCCGGAGATCAGGTCTGAAATTGGTTTTCCTTGACGGTCCCACCAAATGATCCCAAACTTTATGCACACCCTACCATGACCATGGCATGCATGTGGTAGTAGAATTCATACACCACGGCCTCATTTTTGTTGAAATCCAATTAATATCCATGTGTATTACATGTAAGTGAGCATGTGTAAAATTTATTAGATGACTTGAAGGTCAAAACATTAACTTGTCATATATGCAAAAAAATGTGTATTGAAT

>TtuSat14-44

TCTAGTGTAAAATTATTTGTACTAGGTTATAGAGCTAGTTCAAC

>TtuSat15-206

CACCCACATAGCATGTGCGAGAAAGTAGAGAGGGTTACGGCAAAAACTGGATGCACTTCGTGTACAAAACAGACAATCTCTTTCGAAGTATCAGGGTTTCATACGGAAACTCGTCTGTTACAAAGGGATTTCATTTTTTGAACTTATTTGAACTCCATACTTTTTGTGTGTTCAAAATGCACCATTCAAAGCCACATCATCAATTT

>TtuSat16-323

AAAATATAAAACCTTGGAAAACTAGCTTTGTTTGTGCAAGAGATCATGTGTGCCAAAATAGGGGTGATTTGGAGGTGGTCGAAAAAATCATCGCATTCCGGAGGGGCCATTTGGTCTAACTTAAGCCAGTTTTTGAACATACGTGATTTTTTCCACCACCTCCAAATCACCCAAATTTTCTTGTACATGGTGACCCACATGTGCCAAACATGGCTATGAAATAAAATTTGGAAAAATAATATTTTACAATGCCCCCTTGAGGTATAGACCGATGTTTTGACCAGTCAGTCTATAGGTCATTATAAATTCAAAAAAATTCAAA

>TtuSat17-567

GAATGACAAAATTATGACCGTTTTAGTGAACATCTGTCCATTCTATGGCAGTCCTTGAAAGTACTTCATTTTCTCGTGCGCTTTTTGCCTGATTGCCCCAAAATTTCTTGTCCTCTATAGAGGAGATATCGTTTCCACATATATATTGACCGATGCCAGGAATGCCCTTTTGTTCTTGGCTTTCACGCGCATGAGTTACAGCATGCCATCCGGCGAAAACAAGGAGAATAAGGCATATAGCCGCTGGATTTTCTTCAATTTACGGCCAAATGAGATTGCCCGGATCATAGAGCATCTGCAGAAAGGTACGTTTGTCCCTACCAGGGGGGCTAATGTCTATAAGCAACATAATCTCTCGAATTTCCTTCAAAAGGAAGTGCCAATGGGTTCAGAAACTTGTCTGAAAAACAGCGACGAATGATGTTCTTTGACTTTCAGGGCCAAAAAGTCCTGCTTAAGGCTGAATTTGAGGGTACTCCGGGCCTCCTGGCGCAAGCGGGGAGTATTTTCTGGATTCGCGCGCTCGCCGCGAGCAGTTTGAGTACTTGTGGGTCCGACACGGACTCC

>TtuSat18-319

CACGGGCAACAGTGACCATGCAGGCCGCGCACGCCAAAATTTGTCGCGTTCCGACGCTGTACGGATTTTCTATGATTTTTCGCACCGAAAACCCCTAAAACACTGCCCGCACGTGACGCAACTTGCGTTCGTAGTCCGTTTTCGTTCATCTCTGGCGTGCGGTCCCCGGGCGGGTCCCCACACGCGCCGCCGAAAGTTGGGTGCATTCCGGCAAACCGAACTGGCACTTGCTGTGCAACCAGGTGGTTTCGGTATGGGCGGAGGGGACCCTCGGTCGTACTTCTCCCGGTCGCATCCGCCAAAGTCGATCATTTTTTTC

>TtuSat19-72

TCAATCTCAAGATGTTGTGTCGGCTGAGTCTCTTGAAGGTGATCCACCCAGAAGGCGTATGCGGTGACTTCG

>TtuSat20-1590

CACCAGGCGGCCTTCGCCGCGCTCGCCGGGCCGCTGCAGCTGCCATCCATCGCCACCACCGCCCAGCCCTGGCTGCAGTGGCAGCCGCCGCTCCTGGCGGCCTCCGCCGCGCCCGGCGCTCCGCCGCAGCAGCCGTTGCCGCTGCCCGGCGTGACACCGCAGCAGCCGCTGCAGCTGCAGCAGCCACCGCCGGTCAGCTCCGCCGCCCAGTATGGGATGCCCTACGACGGGACTGCGACGACCTCGTTCCCATCAGCGCCGCCGCCATCCCAGGGCGTCCACATCCAGCAGATCAAGTCCCCGCCGTCGCCGTCACCGCTTCCGTCTTGGATCGCTACCCGCCACGTGTCGGCGGCGGTGAGGCTGCAGGCTGCTGCGCGCGGCCTCCTAGCGCGTCGGCGTGTGCGGGAGATGCGTGGTCTGCAGCTGCCGCTCCTCCAAGTTGCCCTTCGCTGCGCAAAGGACCTCGATCTCGTCCGCTGCGTCGGGGATCTTGGGCATGCGGTTTCCCCCACGGGCGGCGGGCATGCTGTTTTCCCCGCGGGCAGCGACCTCAAAGTCTGCGACATCGGCGGTTGGGGGGGCGCACCCCTCCTCGTCATTCTCCATCGCAAGCCCTCCACTCTTCCCTGTGCGGTGCAGACCAACAGCCGTCCGGCGGGGAGAAGGCATGGTGTCACCGACAGCAGCGCACCGCGTAGCACCACTGCATTCCGCCACCGGCCGCCGCGAGGGCGCCTCTGCTGGTCACTCTTGCGACCACTTCCAGGTGGCCATACACATGCACTCCTTTTGTCCAGGTGGTGTCCATGGGATCCAGGTGGCTGTACACGTGCACGTCCGACGTGCGGATGGTGTCCACTTTTTGTTAAGGGGTCCAAAATAAAGCGTCCCAGTCCATTTCAGGTTGAGAGTAATAAAACAAGCCGAGATGTAAAAGGCTTGTTTTTAGGTGTTAGGTTTGTGTTGCGTCGAGTCATGGTTATAAGTTGGTTAGGCTGCAGCTCGAGGACAAGCTGCATGTCCAGGTGGGGTGTAGTGTTAGAGTACGTAATGGGCCTAATGGGCCCATTAGTCTTAGGGTTAATTAGAGATAAGGGTCGCTTGCTTAGGGGTCAAGTAAGCCTTGCTTGGGAGTCAAGTAAACCTCTCTATATAAAGAGAGGAGATGTATCAATCTAATCAAGCAAGAATTAAGAAGGAAATCCCTTCCCTCTTGCCCGGCCGTGGGCAAAAAGGCCCCCGGCCGGCCCTCTCGCGCCCTCCTTCTAGCAGCGCCATAACAATTTGGTATCAGCTAGCTTCGGTTCGATCATGTCTTCACCGCCGCCAAGCCCGTCTCTTCCGCTGCCGGTCACCTTGTCGCCTCCGGCGACCACCACGACCGTCGCCCCGCTCCTGCCCGCGCCGGGATCCTCCGTCGCGCCCGCCCCCGCCGTCCTCACCCCGGAGGAGGTGTCCGGGGTGCTGCGGGACCTAACCCAGGCGGTCCAGGAGATCCACCTGTTCTTGGCCGGGTCCTACGGGCCGCACCCGGCTGCGCCGCCCATCACCGCCACCGCGCCGCCGTGGCTGCCGTGGCAGCCGCCG

>TtuSat21-318

GTAGTCGGGAACCCCCTCCCCGGCAGACCGAAAAGTAGCTGGTTGCACTGGGGCTACGTGATCGCATGCATGCAATTGCACCAAAGTGTGCGTACATGTGGGCACGGCCAACGTAGGCCCCCACGCAAGGTTGGAACGAAAACGGACACAAAACGAACGTTGCGTCACGTCCGGGCAGTGTTTTTGCGATTTTCGACCTGGAAAACCCTAGAAAATGCGTCGAGCGTCGGAAAAATATGCAAATTGGCGTGGGTGCTGTCGATGGTGATGCCAACGTGTGGAAAAAGTTTCGTGCCATTTGGCGGAGTCAAAAAAAAA

>TtuSat22-322

CGGTTCCACCGCACCCGCACCATCCCCCCTCTAAATTACGGCTGCTCTGCCTTCCGTGGAGGGGGGCCACACCACGGAGCCCCAAGACGGGCGTCTACGCATGCCAGAACACTTTCACACATGCATCGGGACCCGTGCGATGTTTCGGTGGCATAGCTACACCCCGAAGGCCACCCCGAATCCCAGTTGACCAGTAGATCTAGCCCTTTGACTTTCGCCGGACGGGCTTTGACCGGTGGACTCTTCCACCTGGTTGCGATAGGTCAGCCCATAGCAACATCCCCGAACACGGTCTGGACCCAACCCAACACTAGATTCCCTC

>TtuSat23-319

CGTGGCATCATGTTGCGTGTCAAAAAAACATGGCGGTGTTTCATGGCTTTGCGAGAGGGAACACCTACTTCACGCATGTAACCCCAGACCGACACCTCAGGTTTGTACTGTGACTATGTGCCAACATGGACGAAACGCACCCAACTTTTGCATGCGCGTGTGAGTGACCCCCACCGGCTCGCACGCCAAATTTCGACGGAATCGGAGTACGAACGGAAGTTGCGTCACGTCGGGAGACCTTTTCTGGGTATTTTCACGGAGAAAATGATAGTAAATGCATCGAGTGTCGAAACGCACCCCAATTTTGCAGGCGTGCTTC

>TtuSat24-889

TAACTATAGCCGGAGAGGCCGATGCATAACTGCGGATGCAACACATAATTGGCATCTCTTGGTTGCACCATGGTGTAAGCATTTCCCAAATAGGTGTTGGTGACATTTAAAACATAATAAATCAGTAATACCACATAATTTGAAGATCGTGATTTACAACAGAAATTGCAAGCTCATGTATACCGAAAAATATAAATGCTTATGGTTGCAATAGAATTAGAAATAACCGAGAGCCTTAAAAAAGGCATGTTGAAAGTGATAACTTGTGATTCCATTGTAGATGTCAAAGTACATCGTCCATGGATGGAGCAGTTAACCTTGCAACCCCCAACAACTATCTGCCTGCACTGTAATATTCTAAGACGATGAAATTCCCATTGAACAATATATACCTGCAATAGTAGATTCTTTTTTTGCAGGAACCTCAGTAGTAGATTCAGCATGTAGCCTAAAAGAAGGAACATAAGACGTCGCTGTCTTGCTGTAAATCAAATTTGCTGATCCACGTGTTGCAAGAAGCCTTGCAACCTATGGCAGAAATACACACAATAATTAAGACAGCCAGCAGAAACAACAGTATTCAGCGACCACATGAATACAATGCCTTGGCGTAGCTACTAAGCATCAGGTCAAGTAAATAGGCAACAAGCCTTGTGTTCCATTGGATTGCATCTAAAGAGTCCTTAACTCCAAAGATTCGAAGAGAAAACAATCAAAATCATGCCTGAAACAAAACACGCAAGAAAAAAGTAAACATGTACCCTGGAGAGGGCGAGCCGGCAAGCCATGAAGGGAATCGCCTGGATTTTTACTCTCGAGGCTCAGCGATTCGTAAGGTCTGCTTCTCCCTGTCTCGCGATAATAATCTCTACTAGAGAGAGAGAGAGAC

>TtuSat25-320

TGCGTTTGGTGCCCGAAGTCGTTCAAATCTTGCGTGGGGCCCTAAGATGGGCATGCCCACTGCCTCCCAAAATTGGGTGACGTTTCGTGCATGCCACCTCGTACCACCAATGCAACCGGCACTGATCGGATATGAACGATGTCAGCCCCGAACTGCGTTCTCTTTTCTTACTCAGCCGGTGGACCCCTAACTTTTTCCACACGCACTCATCGGCAGGGCCATCATGCCTGCAAGTTTTCATAATTTTCCGGCACCGTATGAATTTCCTATGGTTTTCTCCGTGAAACGCACCGAAAACACTGACCGGGCGTGACGCAACG

>TtuSat26-732

TTATCAGATTTCTATAGGTAAGTTGCTATCTGAGGTGCATATATATGCATGGACTACCCTCGAAGCATGAAATGGTGGAAACGATTTCGGAATATGTCGATGACGCTTAATTAATAAGTTTTCAGTTTTGAACGAAGTGAAAATCTATAGTGATGGATAAAAAGAGAACAAAATTGGTGATGAAATTATATGATTACTAGTTATAAGTTGTTATACGAGATCCATATGGTCGAACATACTACTTCTGAAGCTTGAAAGGAAGAAATGCTGGTGGAATGTCTAAATGATGCTATTTAAGTAAGTTTTCTGTTTAGCGAATAAAAAATCATCGGTAACGGATCAAAAGTTTATGTAAATATGCTAAACATGAATAATTTATGTGGATAAATTGTTCTATGACTTTCACATGGCGGTCGAGACTACTCTCGAAGAATGGAATGATGGAAAGGCTGTTATAATGTGTCTAATGTCCTATTTAAATGATTTCTGAGTTTCAAAGGAGGAGAAAATGTAAAGTAATAAATCAAAAGGTTATGAAAAACTACTAAAAGTTATGTGACTCCTACATATATAAGTTGTTGTAGGATTTTCAAATGGCGGCATAGACAACTTCGAAACAAGAAACGAAGCAAGGCTTCTATCACTTGTCGATAATGCTATTTAAACAAGTTTTCAGTTTCGCAAAAATAGAAAATCTACCATGATGGATCAAAACGGTTAAAATGTATCAAA

>TtuSat27-528

GAAAGGGTACAAACAAAATTACTAGTACAAACAAAGGAGTATCACTATAGATAGATGTTGCCGGCCCTAGCTGCATTGATCATAGCTGGTAAATTCAAAATAATTTCTTCAGTTATGATGTAGCATTGATAGATGCCAGGGTGGCTCTCCAGGTTCGGCATTGCTTTGAAGCAACACCACTGATTTGTTTGGCTAGAGCACACTTTCTTGTCAAGAAACCGAATGAACTCCACCTTAAATCATATATTTGGCAGATCTTGTGAAAAATCAAAATCTAAATGGGTAACCATTAATATAACAGAGAGCGTGCACTAAAAGAGGGCCGGGGGAAGCGGAAAGCGTTACAACATTCCACCTTCAGAAGGGTTACAAATGGGCGGCCTTTGCCCGTTTCCAAACTTGCGAATCCGCGAAATCACCCCGCTGCGTGCCGCCTCTAGTATCAGGTTGAAAAAAGATCCGTGTACACGGAGGGAGGAAGTAGAACGGACCCGGTTCCAAACATCCATTTTTACGGTTGAAAAAAAA

>TtuSat28-175

CAAGTTTTTAGTTCACGCAAATCTAGTTCACCAAGTTCAATTTTAGTTCATCAAATCATAATTTTAGTTCACTAGATTCACATTCAGTTCACTACAATTCTCATCATAATCTTAAAGTTTAGTTCACTAAAAAACACCATCTAATTCAGTTCATTAAAATATGTTCATCTGGATT

>TtuSat29-210

TTCCTTTAGTTTGCTCATTCGCTTAATTAGTTACATCGAAGATCTAGACAGAAGGTCCTGTGTTCAATTCCCACAATGTTGATTTATTTTGACCCAATTATTTTTCGCGGTCTCTATGAAAGCCCATAAAAGGCCCATCAACATAGAAGTACCTGGCCCAGATTGACCAAACGAAAAGGACTAAACCAAACCAAGAAAAGGACGCAATCC

>TtuSat30-543

TCCGGAACTCAATTTTACAAGCCGGATCTTGCCTCTGAAATGTTGTCGAAGCTTGCGAGCGAGTTAAGGGCGCATACAACTTTTGGTTTTGATTGTGATTGCGGGTCATGGAGCTCCAATGGAGTTTTTATGGCAAAATTGTGGCCGTTTTATGGATACAACATCGCGGGACAGACCGTGAATACAACTTTCAAGGTAAGTTGATCGCACCAACGAGCCATCTTGCACCATTCGGAATGACCTATAATTTTTCGAGTGCATAAACGGAGTGAGGATGAACTGTTATGTACTTTATGATCCAAGAATAATGCATCCGCTGGTGAAAACGTGAGGGTGGTAGCGGGATGGGTGATAGACCATGTAAAGCATGAATTCTTGGGTTTCGATGCAACGAAGAGCCTCCTCCTTGTGTCGTGACTGAACCTGTAGTCTTTCTAGTTTGTAATGGAAGGATGGTGCATTGTTCTATATGTTATTGTCCATATATCAGTCCGTAGGTGAGCTCATGGAAGGGTGGTAGAGTATACTTTAAGCATAAAATC

>TaeSat31-54

TAAAATACAGTATTGCGATTATCCACCTCAATTTTATATCACTAATTTCAGTAC
