# Supplementary figures and images for: The highly dynamic satellitomes of cultivated wheat species
Source: Ann Bot. 2024 Aug 30;134(6):975–92. doi: 10.1093/aob/mcae132 (PMC11687632; doi:10.1093/aob/mcae132)

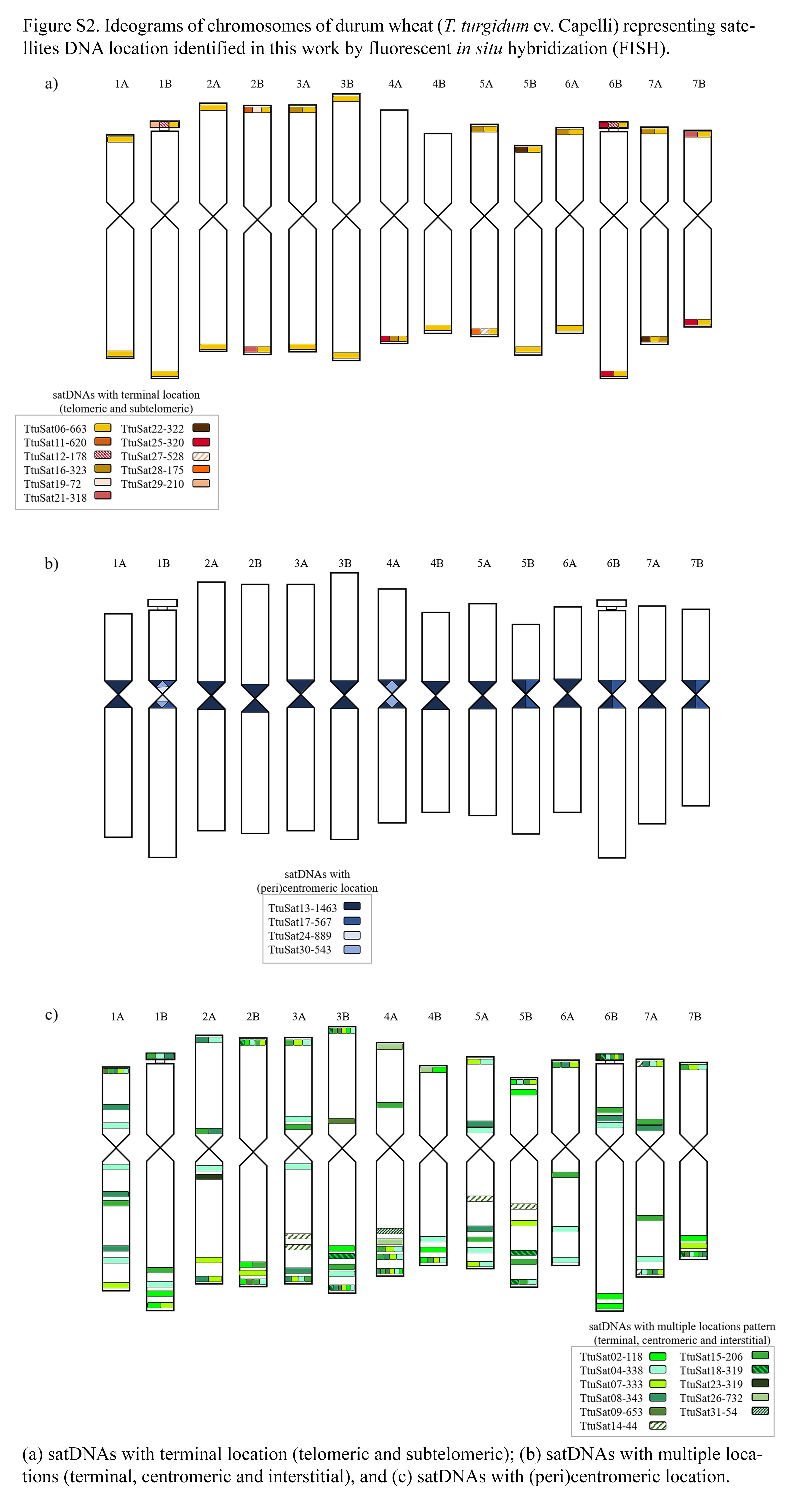

Supplement: mcae132_suppl_Supplementary_Figure_S2 [file mcae132_suppl_supplementary_figure_s2.jpeg]

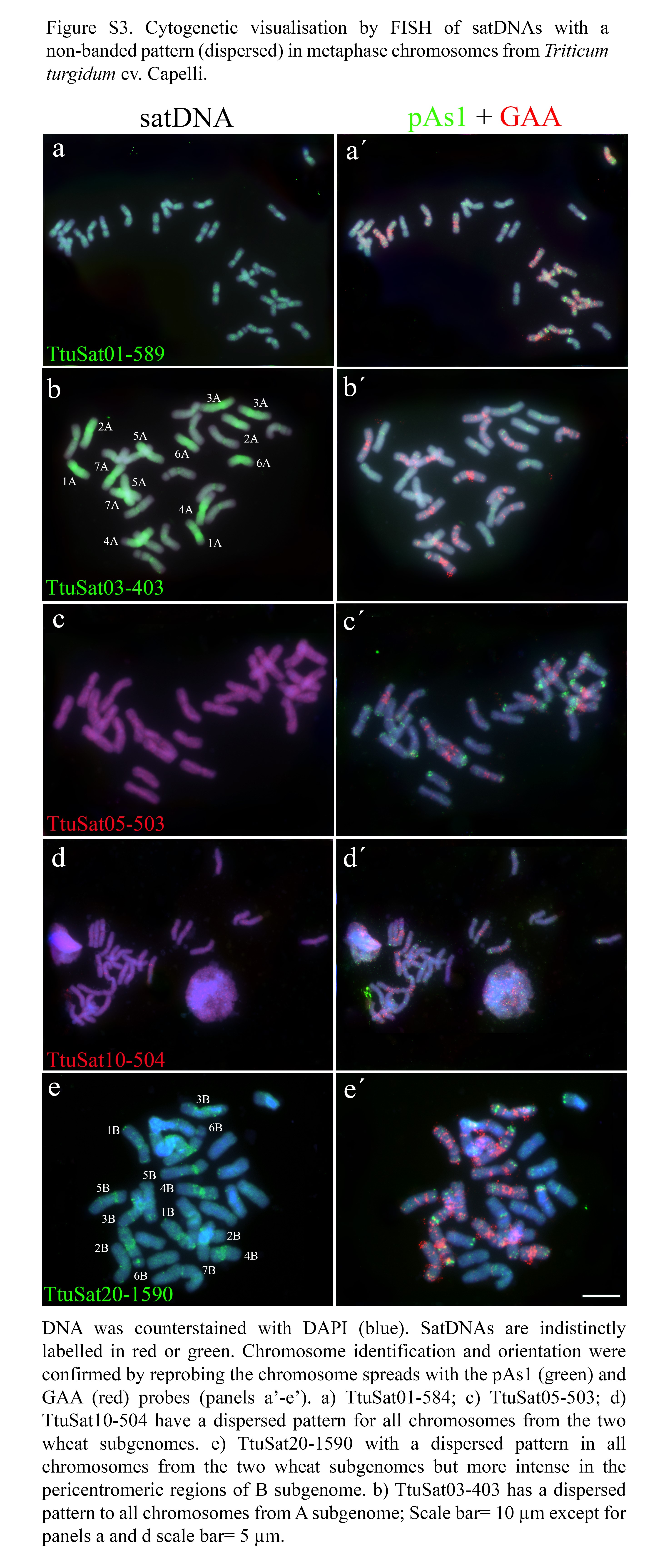

Supplement: mcae132_suppl_Supplementary_Figure_S3 [file mcae132_suppl_supplementary_figure_s3.jpeg]
