# Supplementary material for: The highly dynamic satellitomes of cultivated wheat species
Source: Ann Bot. 2024 Aug 30;134(6):975–92. doi: 10.1093/aob/mcae132 (PMC11687632; doi:10.1093/aob/mcae132)
Supplement: mcae132_suppl_Supplementary_Table_S1 [file mcae132_suppl_supplementary_table_s1.docx]

Table S1. Primer designed in this study to amplify each satDNA family.

| satDNA | Forward | Reverse | T annealing (ºC) |
| --- | --- | --- | --- |
| TtuSat01-589 | CAGAATAACAGGGGGTGTGG | GCCGTAGTGCTTCTTCAACC | 60 |
| TtuSat02-118 | TGGGGTTCGTCAATCATGGA | GGTCATCAACACTCGCAGTT | 55 |
| TtuSat03-403 | TGTTTTGCGGATGGTCAGTA | CATGGCAAGAACAAGTGCAT | 60 |
| TtuSat04-338 | GAGTAGAGAGGGTTACGGCA | TGTGGCTTTGAATGGTGCAT | 55 |
| TtuSat05-503 | AATCACTGCCTCTTTTCGCC | AAACATGCACCCAAGGACAC | 55 |
| TtuSat06-663 | CCAACTGAATCGGCGGAAAT | CAACCACTAGCTAGGAACGGT | 60 |
| TtuSat07-333 | ATATTGCTGTGGGTTTGGCC | GTCCCGGCTAACCCTGTAG | 58 |
| TtuSat08-343 | CGTGTTGGAAATTGATGACG | TGTTGGAACTTGGCATGGTA | 55 |
| TtuSat09-653 | ATGCGTTTTTCGTGAAGCGG | CATCACAAGACGGAGGCATG | 60 |
| TtuSat10-504 | TCATGCCACCAAGATGATGTG | CATGTGCCCATGCCTTGAGA | 55 |
| TtuSat11-620 | CCTTCACAATGCTTCTAGGTGG | CCTCCAAATTGCAGCTCAGTC | 55 |
| TtuSat12-178 | ACTCACATATGGCCGGTTTT | AACACCTCGATAACTTGCTCA | 55 |
| TtuSat13-1463 | TTGGCGAGGTGGGACTAAAC | GGATCATTTGGTGGGACCGT | 55 |
| TtuSat14-44 | GAACTAGCTCTATAAGCTAGT | AACTCTAGTGTAAAATTATTT | 42 |
| TtuSat15-206 | GTTGAGAGGGTTACGGCAAA | GGCTTTGAATGGTGCATTTT | 60 |
| TtuSat16-323 | AGGGGCCATTTGGTCTAACT | TATACCTCAAGGGGGCATTG | 55 |
| TtuSat17-567 | TTTGCCTGATTGCCCCAAAA | TGCTCTATGATCCGGGCAAT | 55 |
| TtuSat18-319 | CGCACCGAAAACCCCTAAAA | ATGATCGACTTTGGCGGATG | 55 |
| TtuSat19-72 | CAATCTCAAGATGTTGTGTCGG | GAAGTCACCACATACGCCTT | 52 |
| TtuSat20-1590 | GACGACCTCGTTCCCATCAG | GGGCAAGAGGGAAGGGATTT | 55 |
| TtuSat21-318 | GCAGACCGAAAAGTAGCTGG | CACCCACGCCAATTTGCATA | 55 |
| TtuSat22-322 | TTTCACACATGCATCGGGAC | TCTAGTGTTGGGTTGGGTCC | 55 |
| TtuSat23-319 | CTTCACGCATGTAACCCCAG | GCAACTTCCGTTCGTACTCC | 60 |
| TtuSat24-889 | GACAGCCAGCAGAAACAACA | AGGCAGATAGTTGTTGGGGG | 60 |
| TtuSat25-320 | TATGAAGGATGTCAGCCCCG | AGTGTTTTCGGTGCGTTTCA | 60 |
| TtuSat26-732 | ATGACTTTCACATGGCGGTC | TTCGAAGTTGTCTATGCCGC | 55 |
| TtuSat27-528 | GAGCGTGCACTAAAAGAGGG | CCGTTCTACTTCCTCCCTCC | 60 |
| TtuSat28-175 | CAAGTTTTTAATTCACGCAAATC | CCAGGTGAACATATTTTAATGAACTG | 60 |
| TtuSat29-210 | GGAGGTCCTGTGTTCAATTCC | GGATTGCGTCCTTTTCTTGGTT | 55 |
| TtuSat30-543 | TTGTGATTGTTTTGGCGGGT | CACCCTCACGTTTTCACCAG | 55 |
| TtuSat31-54 | TAAAATACAGTATTGCGATTAT | GTACTGAAATTAGTGATATAA | 42 |
