# Supplementary material for: The highly dynamic satellitomes of cultivated wheat species
Source: Ann Bot. 2024 Aug 30;134(6):975–92. doi: 10.1093/aob/mcae132 (PMC11687632; doi:10.1093/aob/mcae132)
Supplement: mcae132_suppl_Supplementary_Table_S3 [file mcae132_suppl_supplementary_table_s3.docx]

Table S3. Homology to satDNAs with transposable elements. Different columns indicate the following dates: Smith-Waterman score of the match, complexity adjusted (SW score); Similarity between 2 aligned fragments; Position in the query (begin-end); Strand (transcript strand (+); complementary strand (C)); Matching repeat; Repeat class/family.

*Column **Sim** contains the value of **similarity** between 2 aligned fragments. The similarity is calculated by (Smit *et al*. 2015):

*Sim = match_count / ( alignment_length - query_gap_length - subject_gap_length + gap_count)*
where:

- *match_count* - number of matching base positions in alignment;
- *alignment_length* - length of alignment, which is number of matches + number of mismatches + length of gaps;
- *query_gap_length* - total length of alignment gaps on submitted query sequence;
- *subject_gap_length* - total length of alignment gaps on Repbase library sequence;
- *gap_count* - number of uninterrupted alignment gaps of any length on either query or subject sequences. From a biological point of view one indel, which corresponds to an uninterrupted alignment gap, reflects one event in evolution and should impact the value of similarity in the same way unrelated to its length.

| **Query sequence** | **SW score** | **Similarity** | **Position in query** | | **Strand** | **Matching repeat** | **Repeat class/family** |
| --- | --- | --- | --- | --- | --- | --- | --- |
|  |  |  | **Begin** | **End** |  |  |  |
| TtuSat01-589 | 4951 | 0.9778 | 1 | 589 | + | EnSpm-5_TAe | DNA/EnSpm/CACTA |
| TtuSat03-403 | 1926 | 0.8182 | 27 | 402 | + | Gypsy-17_TAe-LTR | LTR/Gypsy |
| TtuSat04-338 | 2563 | 0.9169 | 2 | 338 | C | EnSpm-N15_TAe | DNA/CMC-EnSpm |
| TtuSat05-503 | 4286 | 0.9762 | 1 | 503 | + | EnSpm-9b_TAe | DNA/CMC-EnSpm |
| TtuSat06-663 | 5434 | 0.9520 | 1 | 663 | + | EnSpm-N1_TA | DNA/CMC-EnSpm |
| TtuSat07-333 | 2437 | 0.9493 | 1 | 333 | + | EnSpm-38_TAe | DNA/CMC-EnSpm |
| TtuSat08-343 | 2663 | 0.9501 | 2 | 342 | + | EnSpm-2_TD | DNA/CMC-EnSpm |
| TtuSat09-653 | 5521 | 0.9844 | 16 | 653 | + | EnSpm-26_TAe | DNA/CMC-EnSpm |
| TtuSat10-504 | 4177 | 0.9663 | 1 | 504 | + | EnSpm-43_TAe | DNA/CMC-EnSpm |
| TtuSat11-620 | 1021 | 0.6772 | 30 | 571 | C | Gypsy-N3_TAe-LTR | LTR/Gypsy |
| TtuSat13-1463 | 11637 | 0.9718 | 1 | 1311 | C | MuDR-45_TAe | DNA/MuDR |
|  | 1340 | 0.9804 | 1312 | 1463 | C | MuDR-45_TAe | DNA/MuDR |
| TtuSat15-206 | 1701 | 0.9469 | 1 | 206 | C | EnSpm-N15_TAe | DNA/CMC-EnSpm |
| TtuSat16-323 | 2415 | 0.9133 | 1 | 322 | C | MuDR-45_TAe | DNA/MuDR |
| TtuSat18-319 | 1826 | 0.8365 | 1 | 318 | + | MuDR-43_TAe | DNA/MuDR |
| TtuSat19-72 | 256 | 0.8333 | 3 | 44 | C | EnSpm3_TM | DNA/CMC-EnSpm |
| TtuSat20-1590 | 6198 | 0.8969 | 1 | 1039 | C | EWAY1_TM-I | LTR/Gypsy |
|  | 1925 | 0.9555 | 1040 | 1285 | C | EWAY1_TM-LTR | LTR/Gypsy |
|  | 1948 | 0.9500 | 1286 | 1590 | C | EWAY1_TM-I | LTR/Gypsy |
| TtuSat21-318 | 2579 | 0.9465 | 1 | 318 | C | MuDR-54_TAe | DNA/MuDR |
| TtuSat22-322 | 2535 | 0.9689 | 1 | 322 | C | EnSpm1_TD | DNA/CMC-EnSpm |
| TtuSat23-319 | 2181 | 0.9094 | 11 | 318 | C | MuDR-51_TAe | DNA/MuDR |
| TtuSat26-732 | 208 | 0.8919 | 287 | 323 | C | HARB-12_FV | DNA/Harbinger |
| TtuSat28-175 | 298 | 0.7349 | 6 | 86 | + | MuDR-26_ZM | DNA/MuDR |
